# When Honesty Meets Modesty: Development of Evaluations on Lying About Achievements

**DOI:** 10.1111/desc.70170

**Published:** 2026-03-16

**Authors:** Shaocong Ma, Eva E. Chen, Michelle Yik

**Affiliations:** ^1^ Division of Social Science Hong Kong University of Science and Technology Hong Kong China; ^2^ College of Education National Tsing Hua University Hsinchu City Taiwan

**Keywords:** lying, modesty, reputation management, social evaluations

## Abstract

Lying about achievements is commonplace in reputation management, yet its social consequences may depend on cultural conventions. The present research examined how Chinese children aged 5–11 years (*N* = 173, 48% girls, *M_age_*: 8.40 years) and adults (*N* = 98, 52% women, *M_age_*: 20.76 years) evaluated truth‐tellers and lie‐tellers who described their achievements in ways that either violated or aligned with the Chinese cultural convention of modesty. When lying violated modesty conventions (Study 1A), both children and adults consistently preferred truth‐tellers over lie‐tellers in evaluations and behavioral preferences; these preferences were unrelated to individuals’ understanding of modesty. When lying aligned with modesty conventions (Study 1B and Study 2), children and adults overall still preferred truth‐tellers over lie‐tellers; however, among individuals who correctly identified the lie‐teller as more modest than the truth‐teller, preferences for truth‐tellers were attenuated. Moreover, when lying signaled modesty, children's age was positively associated with preferences for truth‐tellers only among those who incorrectly identified which protagonist was more modest, but not among children who correctly identified the lie‐teller as more modest. Together, these findings demonstrate that preferences for honesty emerge early and remain stable across development, but are flexibly shaped by modesty conventions and individuals’ understanding of those conventions.

**Summary:**

- Children and adults consistently preferred truth‐tellers over lie‐tellers when lying violated cultural modesty conventions.
- Preferences for honesty remained robust even when lying aligned with modesty conventions, but were attenuated among individuals recognizing modest intent.
- Individuals’ understanding of modesty moderated evaluations of honesty, indicating that cultural conventions shape social evaluations beyond simple truth‐lie distinctions.

## 1 Introduction

*Lying* is defined as acts committed by individuals who know the truth but intentionally provide false information to others in order to control the recipients’ impressions or to impact their beliefs or behaviors (Lee 2013). One common domain of lying is the misrepresentation of personal achievements, which has far‐reaching consequences for individuals’ reputations and social relationships. People can misrepresent their accomplishments to gain approval, avoid punishment, or enhance their social standing. These types of behaviors occur across diverse situations, such as children overstating their performance on challenging games to gain approval from peers (Lobel and Levanon 1988); job candidates exaggerating qualifications to secure employment (Weiss and Feldman 2006); and political figures inflating achievements to attract voters (Walton and Cohen 2007). These examples illustrate the widespread nature of lying and its potential to shape people's reputations and their relationships with others.

Although lying is often deemed morally wrong, its social approval can vary depending on cultural norms and values. In some cultural contexts, certain types of lies—particularly those motivated by *modesty*—are socially tolerated or even encouraged. Modesty functions not only as a moral virtue but also as a communicative norm that guides how individuals present themselves in social interactions, particularly in achievement‐related contexts (Koh and Wang 2012; Watling and Banerjee 2007). Modesty‐related lies, such as intentionally downplaying one's achievements to promote social harmony, are viewed more favorably in cultures that prioritize self‐effacement over self‐promotion (Lee et al. 1997; Schueler 1997). In this research, we define modesty‐related lies as instances in which individuals understate their accomplishments relative to their peers (Fu et al. 2016). This study examined how Chinese individuals evaluate others who lie about achievements that either align with or violate the cultural convention of modesty. By comparing judgments of children (ages 5–11 years) and adults, we also investigated the developmental trajectory of evaluations of honesty and modesty.

### 1.1 Intentional and Conventional Components of Lying

Lying has two essential components: an *intentional* component and a *conventional* component. Lie‐tellers are aware of their own mental states and those of the recipients, so that they can tell lies to *intentionally* change others’ beliefs and behaviors (Lee 2013). For example, in a donation game, German preschoolers exaggerated their contributions when reporting to peers, recognizing that generosity is socially valued and intentionally providing false information to gain approval (Klafka and Liszkowski 2021). Understanding of this intentional component emerges early in development (Bussey 1992, 1999; Rizzo et al. 2019). By 4 years of age, children reliably distinguish truth‐telling from lying, evaluate truth‐tellers more positively, and distrust individuals with a history of deception (Bussey 1992, 1999; Stengelin et al. 2018). With age, children also become increasingly sensitive to the intentions behind false claims. For example, American children aged 3–13 years judged unintentional false claims as less blameworthy than deliberate lies, with older children focusing more on the claimant's benign intentions than on the falsehood itself (Rizzo et al. 2019).

Evaluations of lying also depend on the conventional component, which reflects the socio‐cultural context in which the lie occurs (Lee 2013). This component captures the fact that some lies are more socially acceptable than others, depending on prevailing cultural conventions. For example, while lies that exaggerate personal achievements are typically judged negatively, lies that downplay one's accomplishments are often more approved by others, especially when they serve prosocial purposes—such as protecting others’ feelings (Heyman et al. 2009; Lee et al. 2001).

Although children generally show greater approval of lies told for prosocial than for self‐promotion purpose (Good and Shaw 2022; Lee et al. 2001), cultures differ in the extent to which these types of lies are valued, and such cultural values are transmitted to children through socialization experiences (Cameron et al. 2012; Shohoudi Mojdehi et al. 2021). In Western societies characterized by individualistic cultures emphasizing personal achievement, modesty‐related lies may be approved but are not central to social norms. By contrast, East Asian cultures, such as China, typically place greater value on collectivistic ideals and interpersonal harmony, making modesty‐related lying especially important (Fu et al. 2016; Schueler 1997; Yik et al. 1998, Yik et al. 2023). Specifically, China puts a premium on modesty, which was historically portrayed as a key virtue (Fu and Lee 2024), and this value is explicitly taught through formal education. For example, a parable introduced in the official third‐grade textbook for Chinese language and literature in Mainland China (Chinese Ministry of Education 2018) depicts the consequences of boasting versus modest self‐restraint in an achievement‐relevant context, showing how immodest self‐presentation can lead to social conflict and negative outcomes.

### 1.2 Perceptions and Behaviors Toward Truth‐Tellers and Lie‐Tellers

How individuals evaluate lie‐tellers versus truth‐tellers depends on dimensions underlying their perceptions. Two primary dimensions—*warmth* and *competence*—underlie judgments of other individuals (Fiske et al. 2002). Warmth reflects perceived intentions toward others (e.g., helpful or harmful), while competence reflects the perceived ability to carry out those intentions (e.g., capable or incapable).

Truth‐tellers are generally seen as warmer, as honesty signals trustworthy and prosocial intentions (Bussey 1999). However, when lying serves culturally approved goals—such as expressing modesty—lie‐tellers may also be perceived as warm. In such contexts, modest individuals are often described as moral and sociable (Bond et al. 1982; Fu et al. 2016), which may contribute to higher warmth evaluations. Competence, by contrast, is usually inferred from observable achievements (Cuddy et al. 2008; Fiske 2018), though individuals, especially children, may form more holistic competence judgments that are influenced by perceived warmth (Roussos and Dunham 2016).

Warmth and competence judgments, in turn, guide behavioral preferences (Fiske et al. 2002; Ma et al. 2023; Ma, Cui, et al. 2024). In general, people prefer to socialize with those viewed as warm (Croce and Boseovski 2020; Eisenbruch and Krasnow 2022), and learn from those perceived as competent, warm, or both (Hermes et al. 2015, 2018; Ma, Payir, et al. 2024). Although lying typically reduces perceived trustworthiness, in cultures where modesty is a valued norm, modest lie‐tellers may still be perceived as trustworthy (Ma et al. 2019), leading others to remain willing to interact with them.

### 1.3 Developmental Sensitivity to Modesty‐Related Lies

Although children as young as 5 years can understand honest or deceitful intentions (Bussey 1992, 1999), they typically develop an understanding of modesty in middle childhood. Prior work suggests that by around 8 years of age, children recognize modesty as a social norm and understand its evaluative consequences, which enables them to identify modest behavior and anticipate its social outcomes (Banerjee 2000; Lee et al. 1997; Yoshida et al. 1982).

In cultures that strongly value modesty, such as China, this understanding shapes how children behave and evaluate lying. For example, Chinese children aged 7–11 years sometimes deny their own good deeds to appear modest, and this tendency increases with age (Fu et al. 2016). When evaluating characters who deny their good deeds, older Chinese children in particular view them as modest and morally positive, despite recognizing that such behavior involves lying (Lee et al. 2001). Together, this literature suggests that ages 5–11 years mark a critical period during which children increasingly integrate cultural knowledge about modesty into social evaluations.

### 1.4 The Present Research

The cultural convention of encouraging modesty in Chinese society provides a unique and informative platform on which to investigate how individuals weigh honest intentions against modesty conventions when evaluating others. To capture potential developmental changes in these evaluations, the present research recruited children aged 5–11 years, along with adults as a comparison group.

We ran three studies to address research questions concerning whether Chinese children and adults show systematic preferences between truth‐tellers and lie‐tellers across evaluative (warmth and competence evaluations) and behavioral (socializing and learning preferences) domains, and whether such preferences differ when lying violates versus aligns with modesty conventions. We also examined whether age and individuals’ understanding of honesty and modesty were associated with their preferences. All studies were pre‐registered (Studies 1A and 1B: https://aspredicted.org/wmrn‐5mh8.pdf; Study 2: https://aspredicted.org/cy35cu.pdf), and data and analyses have been made publicly available on the Open Science Framework to ensure transparency (https://osf.io/mf3ya/overview?view_only=ccf5b1c1e9d644bf9f00170b26644560).

## 2 Study 1A

### 2.1 Method

#### 2.1.1 Participants

An a priori power analysis was conducted to determine sample size requirements. In the absence of prior effect size estimates, we assumed medium effects following conventional guidelines (Cohen 1988). For one‐sample *t*‐tests comparing choices against chance (50%), a medium effect size (*d* = 0.50) with 90% power and *α *= 0.05 required 44 participants per age group. A separate regression analysis (*f^2^* = 0.15; 90% power, *α* = 0.05) indicated 73 children were needed to detect age‐related trends among children.

In total, 108 children and 57 college students (all Han Chinese) were recruited from an urban city in China, where modesty is emphasized as a moral value in formal education and everyday socializations (Chinese Ministry of Education 2018). Following pre‐registered criteria, participants who failed three checks for comprehension and memory were excluded from the analyses (three children and eight adults). The final analytic sample included 97 children (47 girls and 50 boys; *M*_age_ = 8.29 years, *SD*_age_ = 1.03 years, age range = 5.79–11.33 years) and 49 adults (28 women and 21 men; *M*_age_ = 21.04 years, *SD*_age_ = 1.09 years, age range = 18–24 years). Parental consent was obtained for all child participants, and adult participants provided informed consent prior to participation.

The child sample (*n* = 97) slightly exceeded the pre‐registered target (*n* = 73) due to the recruitment of an additional group of third‐graders, who would have been learning about modesty in school (Chinese Ministry of Education 2018). No analyses were conducted prior to completing data collection, and main results did not differ between the pre‐registered and full sample. For transparency, we reported findings from the full sample.

#### 2.1.2 Materials

The experiment was administered on a laptop for all participants. Children completed the experiment with the assistance of a trained adult experimenter, who read the instructions and stimuli aloud and recorded their responses. Adults completed the same tasks independently by reading the instructions on the screen.

All participants were introduced to two protagonists who had completed a test involving labeling four different objects. Both protagonists received the same score—two correct answers out of four—while their classmates had answered all four questions correctly. However, the protagonists described their performance differently: The truth‐teller accurately reported getting two questions correct, whereas the lie‐teller falsely claimed to have answered all four questions correctly. To ensure that children fully understood the scenario, they completed a *comprehension check* after the story. Adult participants were not given this check due to their expected proficiency in reading and comprehension.

Next, participants completed a *memory check* to assess their recall of each protagonist's self‐presentation. Those who passed continued to the main tasks, which were presented in randomized order: (a) a *warmth evaluation task*, (b) a *competence evaluation task*, (c) a *socializing preferences task*, and (d) a *learning preferences task*. Following these four main tasks, participants provided (e) *praiseworthiness and likability judgments* of the two protagonists, along with open‐ended explanations. Finally, participants answered questions assessing their (f) *honesty and modesty understanding*.

The protagonists’ gender (male or female characters) and age (child or adult characters) were matched to the participants. To help participants distinguish between the protagonists (truth‐teller or lie‐teller) onscreen, each was paired with a geometric shape (triangle or square), with shape‐protagonist pairings counterbalanced across participants. Similarly, the pairings of protagonist photos with their behaviors (truth‐telling or lying) and the order in which they were introduced were counterbalanced. In the learning preferences task, the assignment of novel object labels to the truth‐teller and the lie‐teller was also counterbalanced. Study materials were chosen based on pilot studies (see Supporting Information) that confirmed protagonists were matched in attractiveness and emotional neutrality, and that novel labels used in our studies were unfamiliar to participants.

#### 2.1.3 Procedure

##### 2.1.3.1 Comprehension Check

After learning about the two protagonists’ performance on the test, children completed a comprehension check question to rate the objective performance of the two protagonists. They were asked: “Compared to their classmates’ performance in the test, do you think they performed quite well or not quite well on the test?” Children who answered incorrectly (“quite well”) received a retelling of the story and were retested; this procedure could occur up to three times. All 97 children responded correctly within two attempts, nine of them required one retelling and then passed the check.

##### 2.1.3.2 Memory Check

Following the protagonists’ self‐presentations, participants were asked to recall the two protagonists’ replies. They were asked two questions: “Do you remember who told other classmates that he/she performed quite well [did not perform quite well]—the child with a triangle or the child with a square?” If a participant answered either question incorrectly, the story was retold and the same two memory questions were administered again; this procedure could occur up to three times. Three children and eight adults failed the memory check after three attempts and were excluded. All remaining participants (97 children, 49 adults) passed the memory check within two attempts; three children required one retelling.

Afterward, participants completed the four main tasks in randomized order to indicate their evaluations and behavioral preferences toward the protagonists. Each task consisted of four items, also presented in randomized order.

##### 2.1.3.3 Warmth Evaluation Task

Participants rated the warmth of the truth‐teller and the lie‐teller on four items by indicating which protagonist better fit descriptors reflecting good intentions toward others (Fiske et al. 2007). The four descriptors were: *friendly, warm, sincere*, and *good‐natured*, adapted from previous research on perceptions of warmth in children and adults (Fiske et al. 2007; Ma et al. 2023). A sample question was: “Which of these two children do you think is friendlier? For example, if you can meet them, who would like to befriend you first?”

##### 2.1.3.4 Competence Evaluation Task

Participants rated competence on four items by indicating which protagonist better fit descriptors reelecting one's capability (Fiske et al. 2007). The four descriptors were: *efficient, intelligent, competent*, and *capable* (Fiske et al. 2007; Ma et al. 2023). A sample question was: “Which of these two children do you think is more efficient? For example, if they both need to complete the same task, who can complete it faster?”

##### 2.1.3.5 Socializing Preferences Task

Adapted from previous research measuring individuals’ willingness to socialize with their peers (Chen et al. 2018, 2025), participants indicated which protagonist they preferred to interact with across four activities: sharing a secret, reading a book, playing (with a toy for children or a video game for adults), and sharing a snack. A sample question was: “If you had a secret, which of the two children would you share it with?”

##### 2.1.3.6 Learning Preferences Task

Adapted from previous research measuring individuals’ willingness to learn from different informants (Chen et al. 2018; Ma et al. 2023), participants viewed four novel objects for which the truth‐teller and lie‐teller provided conflicting labels. Participants indicated which protagonist they preferred to learn from. A sample question was: “The child with a triangle calls this object *woxie*; the child with a square calls this object *tuodie*. Would you prefer to call it *woxie* like the child with a triangle or call it *tuodie* like the child with a square?”

##### 2.1.3.7 Praiseworthiness and Likability Judgments

Next, participants explicitly judged the *praiseworthiness* and *likability* of the truth‐teller and the lie‐teller, and justified their judgments. Sample questions included: “Which one of these two children do you think we should praise more [other people would like more]? Why do you think he/she should be praised [other people would like him/her more]?”

##### 2.1.3.8 Honesty and Modesty Understanding

Finally, participants’ explicit understanding of the protagonists’ behaviors was assessed using two sets of questions targeting honesty and modesty, respectively. For honesty understanding, participants first self‐reported whether they knew the meaning of truth‐telling (“Do you know the meaning of telling the truth?”) and then identified which protagonist did not tell the truth (“Which person do you think did not tell the truth?”). For modesty understanding, participants similarly self‐reported whether they knew the meaning of modesty (“Do you know the meaning of modesty?”) and then identified which protagonist was more modest (“Which person do you think was more modest?”). No corrective feedback was provided regardless of participants’ responses.

### 2.2 Results

#### 2.2.1 Data Analytic Strategy

Although our pre‐registration specified *t*‐tests and ANOVAs, we employed logistic mixed‐effects models (LMMs) to better account for repeated measures, following current methodological recommendations (Muradoglu et al. 2023). Selections of the truth‐teller were coded as “1,” and lie‐teller as “0.” We first fitted an intercept‐only model with a random intercept for participant ID to account for within‐participant clustering. The intercept reflected participants’ overall preference for the truth‐teller versus lie‐teller.

Next, we fitted the main‐effects model, including fixed effects for age group (1 = children [reference level], 2 = adults) and modesty understanding (0 = incorrect understanding [reference level], 1 = correct understanding), both deviation‐coded. Although honesty understanding was preregistered as a predictor, it was excluded from the final models because very few participants in Study 1A answered this question incorrectly, resulting in near‐zero variance and unstable estimates.

To examine age‐related trends within the child sample, we fitted additional LMMs including children's age in years (mean‐centered), modesty understanding, and their interaction, with a random intercept for participant ID. Participant gender was not included because the gender‐matched design minimized potential in‐group gender preferences (e.g., Ma et al. 2023).

Further details about the analyses are provided in the Supporting Information. All analyses were conducted in R version 4.5.2. (R Core Team 2020) and JAMOVI version 2.6 (The Jamovi Project 2025).

#### 2.2.2 Honesty and Modesty Understanding

We report the results for honesty and modesty understanding first as they served as key indicators in subsequent analyses. For both constructs, participants answered two questions: (a) “Do you know the meaning of telling the truth [modesty]?” and (b) “Which person do you think did not tell the truth [was more modest]?” For the former self‐report question, 100% of participants (*N* = 146, including children and adults) reported that they knew the meaning of telling the truth, and 62% of participants (42% of children and 100% of adults) reported that they knew the meaning of modesty.

For the latter identification question, selection of the lie‐teller was coded as correct for honesty understanding (as they did not tell the truth), whereas selecting the truth‐teller was coded as correct for modesty understanding (as they were more modest). Overall, 95% of participants (99% of children and 88% of adults) correctly identified the lie‐teller as not telling the truth, and 90% of participants (88% of children and 96% of adults) correctly identified the truth‐teller as more modest; both rates were significantly above chance (50%), *p*s < 0.001. We used forced‐choice identification questions as primary indicators of honesty and modesty understanding in subsequent analyses, for two reasons. First, participants’ responses to self‐report questions showed limited variance, particularly for honesty. Second, forced‐choice identification questions more directly captured participants’ context‐specific understanding of honesty and modesty.

#### 2.2.3 Warmth Evaluation Task

Overall, children and adults were significantly more likely to select the truth‐teller as being warmer than the lie‐teller, *B_intercept_* = 2.69, *OR* = 14.69, 95% CI [7.90, 27.30], *p *< 0.001. An LMM including fixed effects for age group and modesty understanding revealed no significant main effects on warmth evaluations: age group, *B* = –0.69, *OR* = 0.50, 95% CI [0.22, 1.16], *p_Bonferroni_* = 0.107; modesty understanding, *B* = 0.75, *OR* = 2.12, 95% CI [0.58, 7.80], *p_Bonferroni_* = 0.259. Within the child sample, an LMM examined effects of children's age (in years), modesty understanding, and their interaction; none significantly predicted children's warmth evaluations (Table 1).

**TABLE 1:** Parameter estimates from LMMs predicting children's preferences for the truth‐teller in Study 1A.

	Warmth Evaluation			Competence Evaluation			Socializing Preferences			Learning Preferences		
	*B*	*OR*	*p*	*B*	*OR*	*p*	*B*	*OR*	*p*	*B*	*OR*	*p*
Intercept	2.36	10.61	<0.001	2.01	7.48	<0.001	3.70	40.29	<0.001	1.57	4.82	<0.001
Modesty understanding	0.29	1.34	0.645	−0.03	0.97	0.967	0.22	1.25	0.826	−0.23	0.80	0.653
Age (Years)	0.28	1.33	0.673	0.92	2.50	0.151	1.23	3.43	0.195	0.30	1.35	0.580
Modesty understanding × Age	−0.32	0.73	0.813	−1.41	0.25	0.270	−2.50	0.08	0.194	−0.02	0.98	0.984

*Note*: *N* = 388 observations from 97 child participants. All predictors were mean‐centered.

#### 2.2.4 Competence Evaluation Task

Overall, children and adults were significantly more likely to select the truth‐teller as more competent than the lie‐teller, *B_intercept_* = 1.58, *OR* = 4.87, 95% CI [3.22, 7.37], *p* < 0.001. An LMM including fixed effects for age group and modesty understanding revealed that children were more likely than adults to select the truth‐teller as more competent, *B* = –1.60, *OR* = 0.20, 95% CI [0.10, 0.41], *p_Bonferroni_* < 0.001. Modesty understanding did not significantly predict their evaluations, *B* = 0.49, *OR* = 1.63, 95% CI [0.53, 5.02], *p_Bonferroni_* = 0.396. Within the child sample, age, modesty understanding, and their interaction did not significantly predict competence evaluations (Table 1).

#### 2.2.5 Socializing Preferences Task

Overall, children and adults preferred to socialize with the truth‐teller over the lie‐teller, *B_intercept_* = 2.83, *OR* = 16.87, 95% CI [8.38, 33.95], *p* < 0.001. An LMM including fixed effects for age group and modesty understanding revealed that children were more likely to socialize with the truth‐teller than adults did, *B* = –2.00, *OR* = 0.13, 95% CI [0.06, 0.33], *p_Bonferroni_* < 0.001. Modesty understanding did not significantly predict their socializing preferences, *B* = 1.19, *OR* = 3.30, 95% CI [0.89, 12.27], *p_Bonferroni_* = 0.075. Within the child sample, age, modesty understanding, and their interaction did not significantly predict socializing preferences (Table 1).

#### 2.2.6 Learning Preferences Task

Overall, children and adults preferred to learn from the truth‐teller over the lie‐teller, *B_intercept_* = 1.52, *OR* = 4.56, 95% CI [3.20, 6.51], *p* < 0.001. An LMM including fixed effects for age group and modesty understanding revealed no significant effects on learning preferences: age group, *B* = –0.50, *OR* = 0.61, 95% CI [0.32, 1.14], *p_Bonferroni_* = 0.120; modesty understanding, *B* = –0.26, *OR* = 0.77, 95% CI [0.27, 2.23], *p_Bonferroni_* = 0.635. Within the child sample, age, modesty understanding, and their interaction did not significantly predict learning preferences (Table 1).

#### 2.2.7 Praiseworthiness and Likability Judgments

For each judgment (*praiseworthiness* and *likeability*), we ran proportional tests to compare selections of the truth‐teller against chance ( = 50%) by age group. For praiseworthiness, 99% of children and 88% of adults judged the truth‐teller as more praiseworthy than the lie‐teller, *p*’s < 0.001. For likeability, 99% of children and 82% of adults judged the truth‐teller as more likable than the lie‐teller, *p*’s < 0.001.

### 2.3 Discussion

In Study 1A, we examined how Chinese children and adults evaluated a truth‐teller versus an immodest lie‐teller who attained the same performance but described their performances differently. Across all measures, both age groups showed robust preferences for the truth‐teller: They judged the truth‐teller as warmer and more competent, preferred to socialize with and learn from them, and evaluated them as more praiseworthy and likable. Crucially, in Study 1A, honesty and modesty were aligned—the truth‐teller was also perceived as more modest. This alignment allowed Study 1A to serve as a baseline context in which no trade‐off between honesty and modesty was required. As a result, participants’ preferences demonstrate that both children and adults reliably endorse honesty when it aligns with modesty conventions.

Despite these shared preferences, age‐related differences emerged in the *strength* of preferences for competence evaluations and socializing preferences. Children showed a stronger tendency than adults to prefer the truth‐teller on these dimensions. This pattern suggests that children rely more heavily on honesty cues to form holistic evaluations than adults. Such age differences align with prior work showing that children's evaluations are more global, with positive characteristics exerting broad influence across evaluative domains (Ma et al. 2023; Roussos and Dunham 2016).

Additionally, fewer children than adults reported knowing the meaning of modesty, yet their performance on the modesty identification question was comparable. This pattern suggests that children may show context‐sensitive understanding of modesty before they can explicitly articulate the concept, consistent with prior evidence that behavioral sensitivity often precedes explicit conceptual knowledge in development (e.g., Apperly and Butterfill 2009).

## 3 Study 1B

In Study 1B, we examined Chinese children and adults’ evaluations and preferences for truth‐tellers and lie‐tellers in a context where lying aligned with cultural conventions. Participants were introduced to two protagonists who performed equally on a test: One truthfully reported their performance, while the other lied to downplay their achievement. Since downplaying one's achievement aligns with the cultural value of modesty in Chinese society, such lying would be considered consistent with the cultural convention (Fu et al. 2016; Lee et al. 1997).

### 3.1 Method

#### 3.1.1 Participants

The same participants who completed Study 1A then proceeded to complete Study 1B.

#### 3.1.2 Procedures

The materials and procedure for Study 1B were the same as for Study 1A, with several differences. Two new protagonists were introduced, who answered two of four questions correctly while their peers answered all incorrectly. The truth‐teller accurately reported answering two questions correctly, whereas the lie‐teller falsely claimed to have answered all four questions incorrectly.

The same comprehension and memory check questions were posed. Five children answered the comprehension check incorrectly on their first attempt but passed on their second attempt. One child answered the memory check questions incorrectly on the first attempt but passed on the second attempt; all adults passed the memory check questions on their first attempt. Because all participants passed the checks within two attempts, they were included in the analyses. Consequently, the final analytic sample for Study 1B was identical to that of Study 1A. Participants then completed the same main tasks as in Study 1A, with updated labels and pictures in the learning preferences task.

### 3.2 Results

The data analytic strategy in Study 1B followed that of Study 1A, using LMMs with selections of the truth‐teller coded as 1 and lie‐teller as 0. Unlike Study 1A, honesty understanding showed sufficient variance in Study 1B and was therefore included as a fixed effect alongside modesty understanding and age group in the main‐effects model. Further analytic details are provided in the Supporting Information.

#### 3.2.1 Honesty and Modesty Understanding

In Study 1B, understanding was assessed exclusively through forced‐choice questions in which participants identified which protagonist did not tell the truth (honesty understanding) and which protagonist was more modest (modesty understanding), because the same participants had already self‐reported whether they understood honesty and modesty in Study 1A. Here, selecting the lie‐teller was coded as correct for both honesty and modesty understanding, because lying to downplay one's achievement aligns with modesty conventions. For honesty understanding, 84% of participants (*N* = 146; 86% of children and 82% of adults) correctly selected the lie‐teller, a rate significantly above chance, *p* < 0.001. For modesty understanding, 67% of participants (*N* = 146; 56% of children and 90% of adults) correctly selected the lie‐teller, also significantly above chance, *p* < 0.001.

#### 3.2.2 Warmth Evaluation Task

Overall, children and adults were significantly more likely to select the truth‐teller as warmer than the lie‐teller, *B_intercept_* = 0.61, *OR* = 1.84, 95% CI [1.20, 2.81], *p* = 0.005. To examine predictors of this preference, we fitted an LMM including age group, honesty understanding, and modesty understanding as fixed effects. Age group did not significantly predict preferences for the truth‐teller, *B* = –0.10, *OR* = 0.90, 95% CI [0.44, 1.84], *p_Bonferroni_* = 0.780. However, stronger honesty understanding was significantly associated with stronger preferences for the truth‐teller, *B* = 2.48, *OR* = 11.99, 95% CI [4.49, 32.04], *p_Bonferroni_* < 0.001. By contrast, stronger modesty understanding was significantly associated with reduced preferences for the truth‐teller, *B* = –2.96, *OR* = 0.05, 95% CI [0.02, 0.12], *p_Bonferroni_* < 0.001.

**FIGURE 1:**
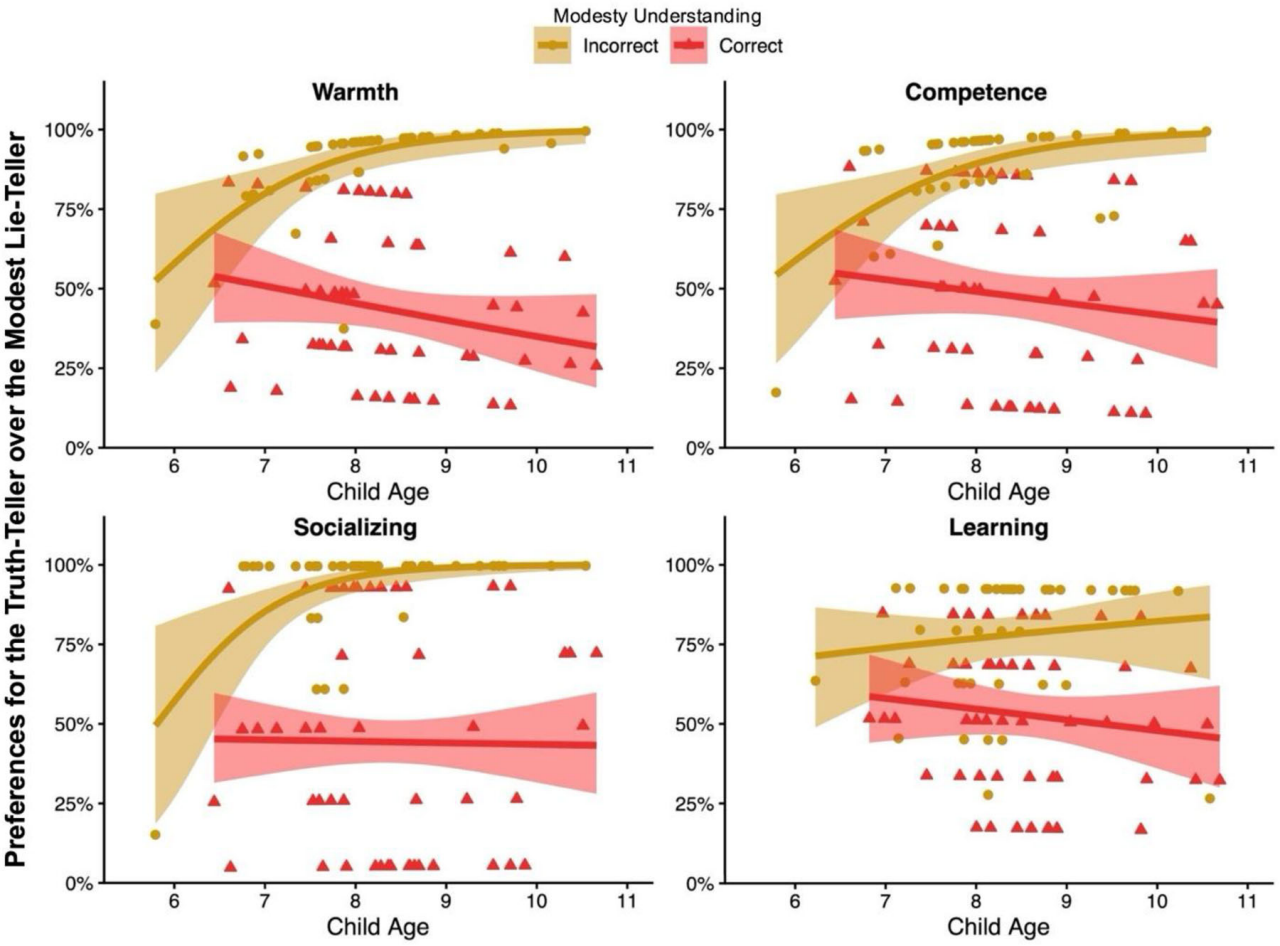
Age‐related changes in children's evaluations of the truth‐teller versus the modest lie‐teller in Study 1B, by modesty understanding. Predicted probabilities (lines with 95% CI) of selecting the truth‐teller over the modest lie‐teller in each task, shown separately by children with incorrect versus correct modesty understanding.

Within the child sample, we examined age‐related trends by including children's age (in years), honesty understanding, modesty understanding, and their interactions as predictors (Table 2). Age alone did not significantly predict warmth evaluations. However, the Age × Modesty Understanding interaction was significant (Figure 1). Simple effects revealed that, among children with incorrect modesty understanding, age was positively associated with preferences for the truth‐teller over the lie‐teller (*B* = 0.98, *OR* = 2.65, 95% CI [1.17, 6.02], *p* = 0.019). By contrast, no age‐related differences emerged among children who correctly understood modesty (*B* = –0.04, *OR* = 0.96, 95% CI [0.52, 1.79], *p* = 0.904).

**TABLE 2:** Parameter estimates from LMMs predicting children's preferences for the truth‐teller in Study 1B.

	Warmth Evaluation			Competence Evaluation			Socializing Preferences			Learning Preferences		
	*B*	*OR*	*p*	*B*	*OR*	*p*	*B*	*OR*	*p*	*B*	*OR*	*p*
Intercept	0.68	1.97	0.032	0.69	1.99	0.052	1.34	3.82	0.031	1.02	2.76	0.003
Honesty understanding	1.77	5.88	0.003	2.04	7.68	0.004	3.40	30.05	0.005	0.01	1.01	0.988
Modesty understanding	−2.87	0.06	<0.001	−2.70	0.07	<0.001	−5.43	0.00	<0.001	−1.64	0.19	<0.001
Age (years)	0.47	1.60	0.107	0.41	1.51	0.227	0.84	2.32	0.156	0.23	1.26	0.427
Honesty understanding × Age	−0.98	0.37	0.080	−0.77	0.46	0.246	−0.99	0.37	0.360	−0.52	0.59	0.351
Modesty understanding × Age	−1.01	0.36	0.027	−1.17	0.31	0.032	−2.20	0.11	0.046	−0.78	0.46	0.077

*Note*: *N* = 388 observations from 97 children. All predictors were mean‐centered.

#### 3.2.3 Competence Evaluation Task

Overall, children and adults were significantly more likely to select the truth‐teller as more competent than the lie‐teller, *B_intercept_* = 0.83, *OR* = 2.29, 95% CI [1.30, 4.06], *p* = 0.004. We then fitted an LMM including age group, honesty understanding, and modesty understanding as fixed effects. Age group did not significantly predict preferences for the truth‐teller, *B* = –0.14, *OR* = 0.87, 95% CI [0.31, 2.45], *p_Bonferroni_* = 0.791. However, stronger honesty understanding was significantly associated with stronger preferences for the truth‐teller, *B* = 2.09, *OR* = 8.10, 95% CI [2.18, 30.40], *p_Bonferroni_* = 0.002. Conversely, stronger modesty understanding was significantly associated with reduced preferences for the truth‐teller, *B* = –3.31, *OR* = 0.04, 95% CI [0.01, 0.12], *p_Bonferroni_* < 0.001.

Within the child sample, an LMM including age, honesty understanding, modesty understanding, and their interactions revealed no main effect of age but a significant Age × Modesty Understanding interaction (Table 2; Figure 1). Age positively predicted preferences for the truth‐teller among children with incorrect modesty understanding (*B* = 1.00, *OR* = 2.72, 95% CI [1.03, 7.22], *p* = 0.044), but not among those with correct modesty understanding (*B* = –0.17, *OR* = 0.84, 95% CI [0.41, 1.74], *p* = 0.640).

#### 3.2.4 Socializing Preferences Task

Overall, children and adults preferred to socialize with the truth‐teller over the lie‐teller, *B_intercept_* = 1.92, *OR* = 6.79, 95% CI [2.42, 19.03], *p* < 0.001. We next fitted an LMM including age group, honesty understanding, and modesty understanding as fixed effects. Age group did not significantly predict preferences for the truth‐teller, *B* = 1.13, *OR* = 3.11, 95% CI [0.64, 15.05], *p_Bonferroni_* = 0.158. However, stronger honesty understanding was significantly associated with stronger preferences for the truth‐teller, *B* = 3.97, *OR* = 53.01, 95% CI [6.99, 401.76], *p_Bonferroni_* < 0.001. Conversely, stronger modesty understanding was significantly associated with reduced preferences for the truth‐teller, *B* = –5.35, *OR* = 0.00, 95% CI [0.00, 0.03], *p_Bonferroni_* < 0.001.

Within the child sample, an LMM including age, honesty understanding, modesty understanding, and their interactions revealed no main effect of age but a significant Age × Modesty Understanding interaction (Table 2; Figure 1). Age positively predicted socializing preferences for the truth‐teller among children with incorrect modesty understanding (*B* = 1.94, *OR* = 6.99, 95% CI [1.00, 48.63], *p* = 0.049), but not among those with correct modesty understanding (*B* = –0.26, *OR* = 0.77, 95% CI [0.25, 2.41], *p* = 0.656).

#### 3.2.5 Learning Preferences Task

Overall, children and adults preferred to learn from the truth‐teller over the lie‐teller, *B_intercept_* = 0.95, *OR* = 2.58, 95% CI [1.65, 4.04], *p* < 0.001. We next fitted an LMM including age group, honesty understanding, and modesty understanding as fixed effects. Age group did not significantly predict preferences for the truth‐teller, *B* = 0.76, *OR* = 2.13, 95% CI [0.84, 5.40], *p_Bonferroni_* = 0.110. Honesty understanding was not significantly associated with their learning preferences either, *B* = 0.65, *OR* = 1.91, 95% CI [0.64, 5.75], *p_Bonferroni_ *= 0.249. However, stronger modesty understanding was significantly associated with reduced learning preferences for the truth‐teller, *B* = –1.93, *OR* = 0.15, 95% CI [0.05, 0.40], *p_Bonferroni_* < 0.001.

Within the child sample, we examined age‐related trends by including children's age, honesty understanding, modesty understanding, and their interactions as predictors (Table 2). Neither age alone nor its interactions with honesty or modesty understanding significantly predicted learning preferences.

#### 3.2.6 Praiseworthiness and Likability Judgments

For praiseworthiness, 69% of children judged the truth‐teller as more praiseworthy than the lie‐teller, significantly above chance (*p* < 0.001), whereas adults did not differ from chance (51%, *p* = 0.999). For likeability, 64% of children judged the truth‐teller as more likable, also above chance (*p* = 0.008), whereas adults again did not differ from chance (37%, *p* = 0.085).

### 3.3 Discussion

In Study 1B, we examined how Chinese children and adults evaluated a truth‐teller versus a lie‐teller whose lying behavior aligned with modesty conventions. Overall, children and adults still preferred the truth‐teller over the modest lie‐teller across all domains. However, their preferences were systematically moderated by individuals’ modesty understanding, revealing the role of culturally grounded conventions in social evaluations.

Honesty understanding robustly predicted stronger preferences for the truth‐teller in warmth, competence, and socializing preferences, consistent with honesty functioning as a default moral criterion. However, stronger modesty understanding systematically attenuated preferences for the truth‐teller, indicating that recognizing modest lying as normatively appropriate leads individuals to evaluate modest lie‐tellers less harshly. Age‐related increases in preferences for the truth‐teller emerged only among children who showed incorrect understanding of modesty. Among children who correctly identified the lie‐teller as more modest, evaluations remained stable across age. These findings suggest that understanding the meaning of modesty may temper an otherwise strengthening valuation of honesty over time.

Finally, praiseworthiness and likeability judgments revealed a developmental divergence in explicit judgments: Children continued to favor truth‐tellers, whereas adults did not. Together, these findings demonstrate that evaluations of lying are shaped by culturally specific conventions, with modesty understanding playing a central role in guiding social evaluations across development.

## 4 Study 2

In Study 1, all participants completed Study 1A before Study 1B, raising the possibility that responses in Study 1B were influenced by prior exposure. To address this concern, Study 2 tested a new sample and examined whether Chinese children and adults would still consider intentions when evaluating truth‐tellers and modest lie‐tellers without prior exposure to immodest lie‐tellers. Study 2 used the same stimuli and procedures as Study 1B, except that participants did not complete Study 1A, and their honesty and modesty understanding were assessed at the end of the study using both self‐report and identification measures.

### 4.1 Method

#### 4.1.1 Participants

We aimed to recruit the same number of adults as in Study 1B (*N* = 49). Because recruiting children is more time‐ and resource‐intensive, we conducted a conservative Monte Carlo power simulation using the *simr* package in R (Green and MacLeod 2016), based on Study 1B child data. The simulation used an intercept‐only LMM of children's learning preferences (yielding the smallest observed *OR*) and indicated that approximately 75 children would provide at least 90% power to detect an effect of comparable magnitude. Data collection was preregistered to stop once this target sample size was reached.

In total, 83 children and 54 college students (all Han Chinese) were recruited from two cities in China. Following pre‐registered criteria and Study 1 exclusion rules, participants who failed the comprehension check (two children) or the memory check (five children and five adults) three times were excluded from analyses. The final analytic sample comprised of 76 children (36 girls and 40 boys; *M*_age_ = 8.54 years, *SD*_age_ = 1.57 years, age range = 5.79–11.16 years) and 49 adults (25 women and 24 men; *M*_age_ = 20.47 years, *SD*_age_ = 1.04 years, age range = 18–25 years). Parental consent was obtained for all child participants, and adult participants provided informed consent prior to participation.

### 4.2 Results

We analyzed our Study 2 data following the pre‐registered plan. For each primary outcome (warmth evaluations, competence evaluations, socializing preferences, and learning preferences), we used LMMs to compare Study 2 with Study 1B. Each model included Study (Study 1B vs. Study 2), age group (children vs. adults), honesty understanding (incorrect vs. correct), and modesty understanding (incorrect vs. correct) as fixed effects, with items nested within participant as random effects. Additional analytic details are provided in the Supporting Information.

#### 4.2.1 Honesty and Modesty Understanding

In Study 2, 100% of participants (*N* = 125) reported understanding honesty, and 68% of those who responded (*n* = 123 as two children did not respond; 49% of responding children and 98% of adults) reported understanding modesty. Consistent with prior studies, participants’ forced‐choice selections were used as the primary indicators of honesty and modesty understanding. Selecting the lie‐teller was coded as correct for both constructs here. When asked to select which protagonist lied, 73% of participants (*N* = 125) responded correctly, significantly above chance, *p* < 0.001. When asked to select which protagonist was more modest, two children responded “I don't know”; for the other 123 participants, 80% correctly selected the lie‐teller, also significantly above chance, *p* < 0.001.

#### 4.2.2 Warmth Evaluation Task

We fitted an LMM with Study (Study 1B vs. Study 2), age group (children vs. adults), honesty understanding (incorrect vs. correct), and modesty understanding (incorrect vs. correct) entered as fixed effects. Results showed a significant main effect of Study, *B* = 0.69, *OR* = 1.99, 95% CI [1.14, 3.48], *p_Bonferroni_ *= 0.015, indicating that participants in Study 2 were more likely than those in Study 1B to evaluate the truth‐teller as warmer than the lie‐teller. Age group did not significantly predict preferences for the truth‐teller, *B* = –0.22, *OR* = 0.80, 95% CI [0.45, 1.43], *p_Bonferroni_* = 0.451. Stronger honesty understanding was significantly associated with stronger preferences for the truth‐teller, *B* = 2.64, *OR* = 14.05, 95% CI [6.77, 19.19], *p_Bonferroni_ *< 0.001. However, stronger modesty understanding was significantly associated with reduced preferences for the truth‐teller, *B* = –2.25, *OR* = 0.11, 95% CI [0.05, 0.21], *p_Bonferroni_ *< 0.001.

Given the significant difference between Study 1B and Study 2, we conducted a follow‐up intercept‐only LMM using Study 2 data to examine overall warmth preferences. Results indicated that, across age groups, participants in Study 2 were significantly more likely to select the truth‐teller as warmer than the lie‐teller, *B* = 0.70, *OR* = 2.01, 95% CI [1.28, 3.16], *p_Bonferroni_* = 0.002.

#### 4.2.3 Competence Evaluation Task

We fitted an LMM with Study, age group, honesty understanding, and modesty understanding as fixed effects. Results indicated no significant effect of Study on competence evaluations, *B* = –0.20, *OR* = 0.82, 95% CI [0.42, 1.59], *p_Bonferroni_* = 0.558. Age group showed a significant effect, *B* = –0.90, *OR* = 0.41, 95% CI [0.20, 0.80], *p_Bonferroni_ *= 0.012, such that, across both studies, children were more likely than adults to select the truth‐teller as more competent than the lie‐teller. Overall, participants’ stronger honesty understanding was significantly associated with stronger preferences for the truth‐teller, *B* = 1.91, *OR* = 6.72, 95% CI [2.93, 15.44], *p_Bonferroni_* < 0.001. Conversely, stronger modesty understanding was significantly associated with weaker preferences for the truth‐teller, *B* = –1.70, *OR* = 0.18, 95% CI [0.08, 0.41], *p_Bonferroni_ *< 0.001.

#### 4.2.4 Socializing Preferences Task

We fitted an LMM with Study, age group, honesty understanding, and modesty understanding as fixed effects. Results revealed no significant effect of Study on socializing preferences, *B* = 0.29, *OR* = 1.33, 95% CI [0.60, 2.98], *p_Bonferroni_ *= 0.482. Age group also showed no significant effect, *B* = 0.21, *OR* = 1.23, 95% CI [0.52, 2.91], *p_Bonferroni_ *= 0.630. However, participants’ stronger honesty understanding was significantly associated with stronger socializing preferences for the truth‐teller, *B* = 3.33, *OR* = 27.99, 95% CI [9.46, 82.84], *p_Bonferroni_ *< 0.001. Conversely, stronger modesty understanding was significantly associated with weaker socializing preferences for the truth‐teller, *B* = –3.29, *OR* = 0.04, 95% CI [0.01, 0.11], *p_Bonferroni_ *< 0.001.

#### 4.2.5 Learning Preferences Task

We fitted an LMM with Study, age group, honesty understanding, and modesty understanding as fixed effects. Results revealed no significant effect of Study on learning preferences, *B* = –0.47, *OR* = 0.62, 95% CI [0.37, 1.05], *p_Bonferroni_ *= 0.075. Age group also showed no significant effect, *B* = 0.18, *OR* = 1.19, 95% CI [0.68, 2.07], *p_Bonferroni_ *= 0.536. However, participants’ stronger honesty understanding was significantly associated with stronger learning preferences for the truth‐teller, *B* = 0.65, *OR* = 1.92, 95% CI [1.02, 3.62], *p_Bonferroni_ *= 0.044. By contrast, stronger modesty understanding was significantly associated with weaker learning preferences for the truth‐teller, *B* = –1.08, *OR* = 0.34, 95% CI [0.18, 0.63], *p_Bonferroni_ *< 0.001.

#### 4.2.6 Praiseworthiness and Likability Judgments

For praiseworthiness, neither children (58%, *p* = 0.207) nor adults (63%, *p* = 0.085) judged the truth‐teller as more praiseworthy than chance. Similarly, for likeability, preferences for the truth‐teller did not differ from chance for either children (59%, *p* = 0.135) or adults (59%, *p* = 0.253).

### 4.3 Discussion

Study 2 examined whether the effects observed from Study 1B were attributed to prior exposure to Study 1A. Using an independent sample, Study 2 largely replicated the key findings from Study 1B, indicating that participants’ evaluations were not driven by order or carryover effects. Across both studies, stronger honesty understanding predicted stronger preferences for truth‐tellers, whereas stronger modesty understanding consistently attenuated these preferences. This pattern supports the interpretation that modesty understanding enables context‐sensitive evaluations rather than a rigid application of honesty‐based rules.

Across competence, socializing, and learning preferences, no reliable differences emerged between Study 1B and Study 2. A study difference emerged only for warmth evaluations: Compared to participants in Study 1B, participants in Study 2 were more likely to view truth‐tellers as being warmer than the lie‐tellers. This difference likely reflects a contrast effect. Specifically, prior exposure to convention‐violating lies in Study 1A may have reduced the salience of honesty as a default cue for warmth in Study 1B, whereas in Study 2—without such prior exposure—honesty functioned as the baseline cue. Importantly, the effects of honesty and modesty understanding were robust across both studies. Overall, Study 2 confirms the robustness of the Study 1B findings and rules out task‐order effects as an alternative explanation of differences between Studies 1A and 1B.

## 5 General Discussion

The present research highlights how cultural conventions shape individuals’ evaluations of lie‐tellers and how these evaluations develop over time. Across three studies (Studies 1A, 1B, and 2), Chinese children and adults evaluated truth‐tellers and lie‐tellers who either exaggerated or downplayed their performance. Overall, participants preferred truth‐tellers over lie‐tellers across evaluative and behavioral domains. However, these preferences were systematically moderated by whether the lie aligned with cultural convention of modesty and by individuals’ understanding of that convention.

When lying violated modesty conventions (Study 1A), preferences for truth‐tellers were strongest across warmth, competence, and socializing and learning preferences. When lying aligned with modesty conventions (Studies 1B and 2), participants still generally preferred truth‐tellers, but this preference was attenuated, especially among individuals who correctly identified lying as modest. This selective attenuation indicates that participants’ evaluations were sensitive to the cultural meaning of the behavior, rather than reflecting a general tolerance for lying. These findings suggest that cultural conventions qualify—but do not override—honesty‐based evaluations.

In contexts where lying serves to signal modesty, understanding of honesty and modesty exerted opposing influences on social evaluations. Stronger honesty understanding predicted stronger preferences for truth‐tellers, while stronger modesty understanding weakened such preferences, reflecting greater tolerance toward modest lying when it was recognized as normatively appropriate. Together, these patterns provide converging evidence that social evaluations are jointly shaped by general honesty standards and culturally specific modesty conventions, contingent on individuals’ recognition of the cultural conventions around the target behavior. This interpretation aligns with prior work showing that culturally conventional forms of lying tend to elicit less negative evaluations (Fu et al. 2011; Lee 2013; Lee et al. 2001; Ma et al. 2019).

Age‐related patterns emerged primarily through differences in cultural understanding. For both children and adults, age group alone usually did not predict preferences once we accounted for their understanding of honesty and modesty. Instead, developmental change was evident in how cultural knowledge modulated evaluations: Among children who had not yet understood modesty, increasing age was associated with stronger preferences for truth‐tellers over modest lie‐tellers, whereas among those who correctly understood modesty, preferences were already attenuated and remained stable across age. This pattern suggests that developmental change reflects the acquisition and application of cultural conventions, rather than a uniform shift in evaluative standards with age.

However, even in contexts where lying aligned with the modesty conventions, participants did not show a definite preference for modest lie‐tellers over truth‐tellers. One possibility is that participants did not uniformly interpret the lie as a clear instance of modesty. Consistent with this interpretation, prior work shows that children can reason flexibly about self‐presentation and recognize multiple motives for self‐disclosure and self‐concealment, including impression management and strategic communication (Hicks et al. 2015). Moreover, the direct comparative nature of our task required participants to weigh honesty and modesty simultaneously, a format known to elicit more deliberative reasoning (Hsee et al. 1999). Under these conditions, honesty may serve as a more direct and salient evaluative anchor, even when modesty conventions culturally allow downplaying one's achievements.

Although the present research provides important insights, several directions remain for future work. First, research should further specify how children engage with modesty conventions by distinguishing among recognition of modest behavior, inference of modest intentions, and endorsement of modesty as a valued virtue, which helps clarify how evaluative use relates to broader internalization across development. Second, extending this work across cultures would help determine which evaluative patterns reflect culturally specific norms versus general socio‐cognitive processes. Third, future studies could refine methodological approaches to capture children's evaluations with greater nuance by allowing non‐forced responses or by incorporating more ecologically valid forms of modest self‐presentation, such as truthfully acknowledging achievements while downplaying personal contribution. Finally, longitudinal or larger‐sample designs would allow finer‐grained tests of developmental change and the role of socialization in shaping modesty understanding over time.

To conclude, this research reveals how cultural conventions and developmental factors impact evaluations of truth‐tellers versus lie‐tellers who exaggerate or downplay their performance. The results suggest that individuals generally prefer truth‐tellers over lie‐tellers. However, preferences for truth‐tellers over lie‐tellers are attenuated when lying aligns with cultural conventions and when individuals develop a mature understanding of those conventions. These findings emphasize the importance of considering the interplay between individual and cultural factors when evaluating lying, which also provides practical guidance for promoting ethical behavior and trust in diverse cultural contexts.

## Conflicts of Interest

The authors declare no conflicts of interest.

## Supporting information




**Supporting File 1**: desc70170‐sup‐0001‐SuppMat.docx

## Data Availability

All studies were preregistered (Studies 1A and 1B: https://aspredicted.org/wmrn‐5mh8.pdf; Study 2: https://aspredicted.org/cy35cu.pdf). Data and analysis scripts are publicly available on the Open Science Framework (https://osf.io/mf3ya/overview?view_only=ccf5b1c1e9d644bf9f00170b26644560).
