# Supplementary material for: When Honesty Meets Modesty: Development of Evaluations on Lying About Achievements
Source: Dev Sci. 2026 Mar 16;29(3):e70170. doi: 10.1111/desc.70170 (PMC12992668; doi:10.1111/desc.70170)
Supplement: Supplementary file 1 — Supporting File 1: desc70170‐sup‐0001‐SuppMat.docx [file DESC-29-e70170-s001.docx]

**Supplementary Material**

**Section A. Study Material**

**Sample of Stories Presented in Study 1A**

[Original Simplified Chinese] 下面我要给你讲一个关于两个小朋友的故事，等你听完后我会问你几个问题，请你仔细地听哦！

[Translated English] I am going to tell you a story about two children, and I will ask you a few questions afterward. Please listen carefully to the story!

这个旁边有三角形的小朋友和这个旁边有正方形的小朋友是同班同学。有一次他们班里有一个考试，要他们说出不同东西的名字。考试中一共有4道题，班里其他的小朋友每道题全都答对了，只有三角形和正方形的小朋友答错了一半的题。

The child with a triangle and the child with a square are classmates. One time, they had a test in their class to name different things. The test had four questions, and all the other children in the class got every question right. Only the child with a triangle and the child with a square got half of the questions wrong.

**Comprehension Check**

你觉得和班里其他小朋友比，三角形和正方形小朋友在考试中表现得不太好还是挺好的呢？

Compared to their classmates’ performance in the test, do you think the child with a triangle and the child with a square performed not quite well or quite well on the test?

我们继续来听故事。当班里其他小朋友问三角形的小朋友在考试里表现得怎么样时，三角形的小朋友说：“我表现得挺好的，我没有答错题。”当其他小朋友问正方形的小朋友在考试里表现得怎么样，正方形的小朋友说：“我表现得不太好，我答错了一半的题。”

Let’s continue with the story. When the other children in the class asked the child with a triangle how he/she performed on the test, the child with a triangle said, “I performed quite well. I did not get any questions wrong.” When the other children asked the child with a square how he/she performed on the test, the child with a square said, “I did not perform quite well. I got half of the questions wrong.”

**Memory Check**

你还记得是哪个小朋友告诉别人说他/她表现的挺好的吗——是旁边有三角形的小朋友还是旁边有正方形的小朋友呢？

Do you remember who told other classmates that he/she performed quite well—the child with a triangle or the child with a square?

你还记得是哪个小朋友告诉别人说他/她表现的不太好吗——是旁边有三角形的小朋友还是旁边有正方形的小朋友呢？

Do you remember who told other classmates that he/she did not perform quite well—the child with a triangle or the child with a square?

**Sample of Stories Presented in Study 1B & Study 2**

下面我要给你讲一个关于另外两个小朋友的故事，这次不是之前故事里的小朋友了。等你听完后我会问你几个问题，请你仔细地听哦！

Now, I will tell you a story about another two children, not the ones from the last story. I will ask you a few questions afterward. Please listen carefully to the story!

这个旁边有三角形的小朋友和这个旁边有正方形的小朋友是班上其他两位同学。有一次他们班里有一个考试，要他们说出不同东西的名字。考试中一共有4道题，班里其他的小朋友每道题全都答错了，只有三角形和正方形的小朋友答对了一半的题。

The child with a triangle and the child with a square are classmates. One time, they had a test in their class to name different things. The test had four questions, and all the other children in the class got every question wrong. Only the child with a triangle and the child with a square got half of the questions right.

**Comprehension Check**

你觉得和班里其他小朋友比，三角形和正方形小朋友在考试中表现得挺好的还是不太好呢？

Compared to their classmates’ performance in the test, do you think they performed quite well or not quite well on the test?

我们继续来听故事。当班里其他小朋友问三角形的小朋友在考试里表现得怎么样时，三角形的小朋友说：“我表现得挺好的，我答对了一半的题。”当其他小朋友问正方形的小朋友在考试里表现得怎么样，正方形的小朋友说：“我表现得不太好，我没有答对题。”

Let’s continue with the story. When the other children in the class asked the child with a triangle how he/she performed on the test, the child with a triangle said, “I performed quite well. I got half of the questions correct.” When the other children asked the child with a square how he/she performed on the test, the child with a square said, “I did not perform quite well. I did not get any questions correct.”

**Memory Check**

你还记得是哪个小朋友告诉别人说他/她表现的挺好的吗——是旁边有三角形的小朋友还是旁边有正方形的小朋友呢？

Do you remember who told other classmates that he/she performed quite well—the child with a triangle or the child with a square?

你还记得是哪个小朋友告诉别人说他/她表现的不太好吗——是旁边有三角形的小朋友还是旁边有正方形的小朋友呢？

Do you remember who told other classmates that he/she did not perform quite well—the child with a triangle or the child with a square?

**Pilot Studies**

Two pilot studies were conducted prior to the current studies to select appropriate study stimuli. The first pilot study was used to select face photos of the protagonists, and the other to select study materials used in the learning preferences task. First, 64 pictures of Chinese faces were generated by artificial intelligence from the Generated Photos website (Generative Adversarial Network, 2018), including 32 children’s photos (16 girls and 16 boys) and 32 adults (16 women and 16 men). In the first pilot study, 102 Chinese adults (54 women and 48 men; *M*_age_ = 24.56 years, *SD*_age_ = 8.27 years) rated (a) emotion neutrality and (b) physical attractiveness of these faces in the photos. Participants were instructed to identify the faces they perceived as (a) having different emotions from the other faces or (b) more attractive or less attractive than the other faces. Based on this pilot study results, 16 face pictures (4 photos for each group of girls, boys, women, and men) were selected for this study. All of the faces were rated as neutral in terms of emotion and physical attractiveness by more than 90% of the participants.

Second, another 86 Chinese adults (41 women and 45 men, *M*_age_ = 26.99 years, *SD*_age_ = 5.71 years) were asked to identify any labels they had known from a list of 13 pairs of novel labels that were prepared for the learning preferences task. These were novel labels adapted from a novel name database (Horst & Hout, 2016) and from previous studies using similar study tasks (Chen et al., 2018; S. Ma et al., 2022). Eight pairs of novel labels that were unfamiliar to all of the participants investigated were selected as the study stimuli. These eight pairs of novel labels together with the pictures of these novel labels are shown in Table A1.

**Table A1**

*Novel Object Pictures and Labels in Learning Preferences Task*

| Study 1A | | | Study 1B & Study 2 | | |
| --- | --- | --- | --- | --- | --- |
| Novel Object | Label 1 | Label 2 | Novel Object | Label 1 | Label 2 |
| 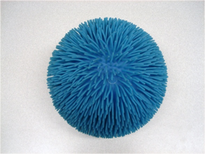 | woxie  (窝协) | tuodie  (拖碟) | 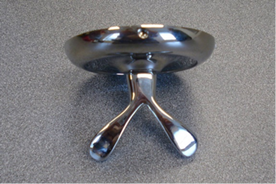 | bosa  (波萨) | tosa  (拖萨) |
| 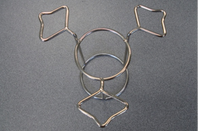 | kita  (凯塌) | mota  (莫塌) | 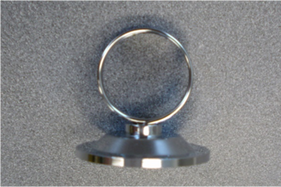 | bido  (⽐多) | deta  (得踏) |
| 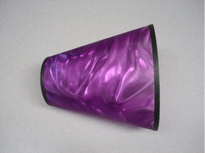 | soobe  (素被) | toobe  (突被) | 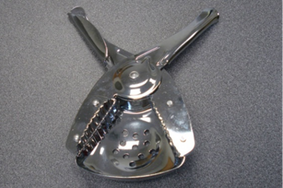 | zeb  (在倍) | zev  (在微) |
| 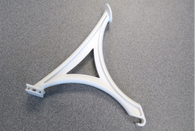 | tanla  (坦拉) | tana  (塔纳) | 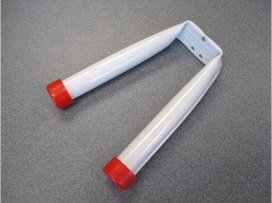 | jiatie  (夹铁) | chitie  (卡铁) |

**Section B. Additional Analyses**

**Study 1A**

**Warmth Evaluation Task**

In the Warmth Evaluation Task, participants’ choices of the truth-telling protagonist (coded as 1) versus the lie-telling protagonist (coded as 0) were analyzed using generalized linear mixed models (LMMs) with a binomial distribution and logit link. All models included a random intercept for participant ID to account for the clustering of responses across items.

We fitted three nested models. First, we estimated a null (intercept-only) model, which captured the overall marginal preference for the truth-telling protagonist across the sample (reported in the main text). Next, we added fixed main effects for age group (children vs. adults) and modesty understanding (incorrect vs. correct). All predictors were deviation-coded (simple coding), ensuring that the model intercept represented the predicted log-odds of preferring the truth-telling protagonist at the average level of each predictor. This main-effects model is also reported in the main text.

A likelihood-ratio test showed that the main-effects model did not provide a significantly better fit than the null model (Δ*LL* = 1.65, *Δdf* = 2, χ² = 3.29, *p* = .193). Detailed fit indices for these models are presented in Table B1.1.

**Table B1.1**

*Model Fit Indices and Comparisons (Null vs. Main-Effects Model) for Warmth Evaluations*

| Model | LogLikelihood | AIC | BIC | Deviance | Residual *df* | Conditional *R²* | Marginal *R²* | LRT χ² (*df*) | *p* |
| --- | --- | --- | --- | --- | --- | --- | --- | --- | --- |
| Null | -219.38 | 442.75 | 451.49 | 438.75 | 582 | 0.47 | 0.00 | — | — |
| Main-Effects | -217.73 | 443.46 | 460.94 | 435.46 | 580 | 0.45 | 0.02 | 3.29 (2) | .193 |

Next, we tested a more complex interaction model that retained the main effects and added the interaction between age group and modesty understanding. Parameter estimates for this model are provided in Table B1.2.

**Table B1.2**

*Fixed-Effects Parameter Estimates for the Interaction Model for Warmth Evaluations*

|  | Estimate *(B)* | *SE* | *OR* | 95% CI  Lower | 95% CI  Upper | *z* | *p* |
| --- | --- | --- | --- | --- | --- | --- | --- |
| Intercept | 1.91 | 0.46 | 6.77 | 2.73 | 16.79 | 4.13 | < .001 |
| Age Group | -1.51 | 0.87 | 0.22 | 0.04 | 1.22 | -1.73 | .083 |
| Modesty Understanding | 1.36 | 0.88 | 3.91 | 0.70 | 21.88 | 1.55 | .121 |
| Age Group × Modesty Understanding | 1.89 | 1.75 | 6.65 | 0.22 | 204.80 | 1.08 | .279 |

A likelihood-ratio test indicated that adding the interaction did not significantly improve model fit over the main-effects model (Δ*LL* = 0.59, Δ*df* = 1, χ² = 1.18, *p* = .277). Detailed fit indices for this comparison are presented in Table B1.3.

**Table B1.3**

*Model Fit Indices and Comparisons (Main-Effects vs. Interaction Model) for Warmth Evaluations*

| Model | LogLikelihood | AIC | BIC | Deviance | Residual *df* | Conditional *R²* | Marginal *R²* | LRT χ² (*df*) | *p* |
| --- | --- | --- | --- | --- | --- | --- | --- | --- | --- |
| Main-Effects | -217.73 | 443.46 | 460.94 | 435.46 | 580 | 0.45 | 0.02 | — | — |
| Interaction | -217.14 | 444.28 | 466.13 | 434.28 | 579 | 0.45 | 0.03 | 1.18 (1) | .277 |

Because the interaction did not contribute to better fit, the more parsimonious main-effects model was selected as the optimal model and is the focus of interpretation in the main text.

To explore age-related trends within children, we fitted another LMM on the child subsample (*N* = 388 observations from 97 participants). The model included fixed effects for age in years (continuous, mean-centered), modesty understanding (incorrect vs. correct), and their interaction (random intercept for participant ID). The full model did not provide a significantly better fit than the null model (Δ*LL* = 0.42, Δ*df* = 3, χ² = 0.83, *p* = .841), indicating limited evidence for age-related effects. Detailed fit indices for these models are presented in Table B1.4.

**Table B1.4**

*Model Fit Indices and Comparisons (Null vs. Full Model) – Warmth Evaluations (Children Only)*

| Model | LogLikelihood | AIC | BIC | Deviance | Residual *df* | Conditional *R²* | Marginal *R²* | LRT χ² (*df*) | *p* |
| --- | --- | --- | --- | --- | --- | --- | --- | --- | --- |
| Null | -135.95 | 275.91 | 283.83 | 271.91 | 386 | 0.27 | 0.00 | — | — |
| Full | -135.54 | 281.07 | 300.88 | 271.07 | 383 | 0.27 | 0.01 | 0.83 (3) | .841 |

**Competence Evaluation Task**

In the Competence Evaluation Task, participants’ choices of the truth-telling protagonist (coded as 1) versus the lie-telling protagonist (coded as 0) were analyzed using LMMs with a binomial distribution and logit link. All models included a random intercept for participant ID to account for the clustering of responses across items.

We fitted three nested models. First, we estimated a null (intercept-only) model, which captured the overall marginal preference for the truth-telling protagonist across the sample (reported in the main text). Next, we added fixed main effects for age group (children vs. adults) and modesty understanding (incorrect vs. correct). All predictors were deviation-coded (simple coding), ensuring that the model intercept represented the predicted log-odds of preferring the truth-telling protagonist at the average level of each predictor. This main-effects model is also reported in the main text.

A likelihood-ratio test showed that the main-effects model provided a significantly better fit than the null model (Δ*LL* = 10.12, *Δdf* = 2, χ² = 20.24, *p* < .001). Detailed fit indices for these models are presented in Table B1.5.

**Table B1.5**

*Model Fit Indices and Comparisons (Null vs. Main-Effects Model) for Competence Evaluations*

| Model | LogLikelihood | AIC | BIC | Deviance | Residual *df* | Conditional *R²* | Marginal *R²* | LRT χ² (*df*) | *p* |
| --- | --- | --- | --- | --- | --- | --- | --- | --- | --- |
| Null | -305.37 | 614.75 | 623.49 | 610.75 | 582 | 0.44 | 0.00 | — | — |
| Main-Effects | -295.25 | 598.51 | 615.99 | 590.51 | 580 | 0.44 | 0.10 | 20.24 (2) | < .001 |

Next, we tested a more complex interaction model that retained the main effects and added the interaction between age group and modesty understanding. Parameter estimates for this model are provided in Table B1.6.

**Table B1.6**

*Fixed-Effects Parameter Estimates for the Interaction Model for Competence Evaluations*

|  | Estimate *(B)* | *SE* | *OR* | 95% CI  Lower | 95% CI  Upper | *z* | *p* |
| --- | --- | --- | --- | --- | --- | --- | --- |
| Intercept | 0.93 | 0.39 | 2.53 | 1.19 | 5.38 | 2.41 | .016 |
| Age Group | -2.08 | 0.78 | 0.13 | 0.03 | 0.58 | -2.67 | .008 |
| Modesty Understanding | 0.84 | 0.77 | 2.31 | 0.52 | 10.39 | 1.09 | .274 |
| Age Group × Modesty Understanding | 1.08 | 1.53 | 2.96 | 0.15 | 59.27 | 0.71 | .478 |

A likelihood-ratio test indicated that adding the interaction did not significantly improve model fit over the main-effects model (Δ*LL* = 0.26, Δ*df* = 1, χ² = 0.51, *p* = .475). Detailed fit indices for this comparison are presented in Table B1.7.

**Table B1.7**

*Model Fit Indices and Comparisons (Main-Effects vs. Interaction Model) for Competence Evaluations*

| Model | LogLikelihood | AIC | BIC | Deviance | Residual *df* | Conditional *R²* | Marginal *R²* | LRT χ² (*df*) | *p* |
| --- | --- | --- | --- | --- | --- | --- | --- | --- | --- |
| Main-Effects | -295.25 | 598.51 | 615.99 | 590.51 | 580 | 0.44 | 0.10 | — | — |
| Interaction | -295.00 | 600.00 | 621.85 | 590.00 | 579 | 0.44 | 0.10 | 0.51 (1) | .475 |

Because the interaction did not contribute to better fit, the more parsimonious main-effects model was selected as the optimal model and is the focus of interpretation in the main text.

To explore age-related trends within children, we fitted another LMM on the child subsample (*N* = 388 observations from 97 participants). The model included fixed effects for age in years (continuous, mean-centered), modesty understanding (incorrect vs. correct), and their interaction (random intercept for participant ID). The full model did not provide a significantly better fit than the null model (Δ*LL* = 1.54, Δ*df* = 3, χ² = 3.07, *p* = .381), indicating limited evidence for age-related effects. Detailed fit indices for these models are presented in Table B1.8.

**Table B1.8**

*Model Fit Indices and Comparisons (Null vs. Full Model) – Competence Evaluations (Children Only)*

| Model | LogLikelihood | AIC | BIC | Deviance | Residual *df* | Conditional *R²* | Marginal *R²* | LRT χ² (*df*) | *p* |
| --- | --- | --- | --- | --- | --- | --- | --- | --- | --- |
| Null | -174.76 | 353.52 | 361.44 | 349.52 | 386 | 0.31 | 0.00 | — | — |
| Full | -173.22 | 356.44 | 376.25 | 346.44 | 383 | 0.32 | 0.02 | 3.07 (3) | .381 |

**Socializing Preferences Task**

In the Socializing Preferences Task, participants’ choices of the truth-telling protagonist (coded as 1) versus the lie-telling protagonist (coded as 0) were analyzed using LMMs with a binomial distribution and logit link. All models included a random intercept for participant ID to account for the clustering of responses across items.

We fitted three nested models. First, we estimated a null (intercept-only) model, which captured the overall marginal preference for the truth-telling protagonist across the sample (reported in the main text). Next, we added fixed main effects for age group (children vs. adults) and modesty understanding (incorrect vs. correct). All predictors were deviation-coded (simple coding), ensuring that the model intercept represented the predicted log-odds of preferring the truth-telling protagonist at the average level of each predictor. This main-effects model is also reported in the main text.

A likelihood-ratio test showed that the main-effects model provided a significantly better fit than the null model (Δ*LL* = 11.45, *Δdf* = 2, χ² = 22.89, *p* < .001). Detailed fit indices for these models are presented in Table B1.9.

**Table B1.9**

*Model Fit Indices and Comparisons (Null vs. Main-Effects Model) for Socializing Preferences*

| Model | LogLikelihood | AIC | BIC | Deviance | Residual *df* | Conditional *R²* | Marginal *R²* | LRT χ² (*df*) | *p* |
| --- | --- | --- | --- | --- | --- | --- | --- | --- | --- |
| Null | -219.89 | 443.78 | 452.52 | 439.78 | 582 | 0.53 | 0.00 | — | — |
| Main-Effects | -208.44 | 424.88 | 442.36 | 416.88 | 580 | 0.51 | 0.14 | 22.89 (2) | < .001 |

Next, we tested a more complex interaction model that retained the main effects and added the interaction between age group and modesty understanding. Parameter estimates for this model are provided in Table B1.10.

**Table B1.10**

*Fixed-Effects Parameter Estimates for the Interaction Model for Socializing Preferences*

|  | Estimate *(B)* | *SE* | *OR* | 95% CI  Lower | 95% CI  Upper | *z* | *p* |
| --- | --- | --- | --- | --- | --- | --- | --- |
| Intercept | 1.59 | 0.44 | 4.91 | 2.07 | 11.64 | 3.61 | < .001 |
| Age Group | -2.93 | 0.85 | 0.05 | 0.01 | 0.28 | -3.45 | < .001 |
| Modesty Understanding | 1.81 | 0.83 | 6.12 | 1.20 | 31.24 | 2.18 | .029 |
| Age Group × Modesty Understanding | 2.21 | 1.65 | 9.13 | 0.36 | 232.94 | 1.34 | .181 |

A likelihood-ratio test indicated that adding the interaction did not significantly improve model fit over the main-effects model (Δ*LL* = 0.92, Δ*df* = 1, χ² = 1.84, *p* = .175). Detailed fit indices for this comparison are presented in Table B1.11.

**Table B1.11**

*Model Fit Indices and Comparisons (Main-Effects vs. Interaction Model) for Socializing Preferences*

| Model | LogLikelihood | AIC | BIC | Deviance | Residual *df* | Conditional *R²* | Marginal *R²* | LRT χ² (*df*) | *p* |
| --- | --- | --- | --- | --- | --- | --- | --- | --- | --- |
| Main-Effects | -208.44 | 424.88 | 442.36 | 416.88 | 580 | 0.51 | 0.14 | — | — |
| Interaction | -207.52 | 425.05 | 446.90 | 415.05 | 579 | 0.50 | 0.14 | 1.84 (1) | .175 |

Because the interaction did not contribute to better fit, the more parsimonious main-effects model was selected as the optimal model and is the focus of interpretation in the main text.

To explore age-related trends within children, we fitted another LMM on the child subsample (*N* = 388 observations from 97 participants). The model included fixed effects for age in years (continuous, mean-centered), modesty understanding (incorrect vs. correct), and their interaction (random intercept for participant ID). The full model did not provide a significantly better fit than the null model (Δ*LL* = 1.30, Δ*df* = 3, χ² = 2.60, *p* = .458), indicating limited evidence for age-related effects. Detailed fit indices for this comparison are presented in Table B1.12.

**Table B1.12**

*Model Fit Indices and Comparisons (Null vs. Full Model) – Socializing Preferences (Children Only)*

| Model | LogLikelihood | AIC | BIC | Deviance | Residual *df* | Conditional *R²* | Marginal *R²* | LRT χ² (*df*) | *p* |
| --- | --- | --- | --- | --- | --- | --- | --- | --- | --- |
| Null | -103.00 | 210.00 | 217.92 | 206.00 | 386 | 0.56 | 0.00 | — | — |
| Full | -101.70 | 213.40 | 233.20 | 203.40 | 383 | 0.55 | 0.02 | 2.60 (3) | .458 |

**Learning Preferences Task**

In the Learning Preferences Task, participants’ choices of the truth-telling protagonist (coded as 1) versus the lie-telling protagonist (coded as 0) were analyzed using LMMs with a binomial distribution and logit link. All models included a random intercept for participant ID to account for the clustering of responses across items.

We fitted three nested models. First, we estimated a null (intercept-only) model, which captured the overall marginal preference for the truth-telling protagonist across the sample (reported in the main text). Next, we added fixed main effects for age group (children vs. adults) and modesty understanding (incorrect vs. correct). All predictors were deviation-coded (simple coding), ensuring that the model intercept represented the predicted log-odds of preferring the truth-telling protagonist at the average level of each predictor. This main-effects model is also reported in the main text.

A likelihood-ratio test showed that the main-effects model did not provide a significantly better fit than the null model (Δ*LL* = 1.42, *Δdf* = 2, χ² = 2.84, *p* = .241). Detailed fit indices for these models are presented in Table B1.13.

**Table B1.13**

*Model Fit Indices and Comparisons (Null vs. Main-Effects Model) for Learning Preferences*

| Model | LogLikelihood | AIC | BIC | Deviance | Residual *df* | Conditional *R²* | Marginal *R²* | LRT χ² (*df*) | *p* |
| --- | --- | --- | --- | --- | --- | --- | --- | --- | --- |
| Null | -307.42 | 618.85 | 627.59 | 614.85 | 582 | 0.32 | 0.00 | — | — |
| Main-Effects | -306.00 | 620.00 | 637.48 | 612.00 | 580 | 0.32 | 0.01 | 2.84 (2) | .241 |

Next, we tested a more complex interaction model that retained the main effects and added the interaction between age group and modesty understanding. Parameter estimates for this model are provided in Table B1.14.

**Table B1.14**

*Fixed-Effects Parameter Estimates for the Interaction Model for Learning Preferences*

|  | Estimate *(B)* | *SE* | *OR* | 95% CI  Lower | 95% CI  Upper | *z* | *p* |
| --- | --- | --- | --- | --- | --- | --- | --- |
| Intercept | 1.53 | 0.37 | 4.63 | 2.24 | 9.60 | 4.13 | < .001 |
| Age Group | -0.49 | 0.72 | 0.61 | 0.15 | 2.50 | -0.68 | .495 |
| Modesty Understanding | -0.26 | 0.72 | 0.77 | 0.19 | 3.13 | -0.37 | .713 |
| Age Group × Modesty Understanding | -0.02 | 1.43 | 0.98 | 0.06 | 16.23 | -0.02 | .988 |

A likelihood-ratio test indicated that adding the interaction did not significantly improve model fit over the main-effects model (Δ*LL* = 0.00, Δ*df* = 1, χ² = 0.00, *p* = .988). Detailed fit indices for this comparison are presented in Table B1.15.

**Table B1.15**

*Model Fit Indices and Comparisons (Main-Effects vs. Interaction Model) for Learning Preferences*

| Model | LogLikelihood | AIC | BIC | Deviance | Residual *df* | Conditional *R²* | Marginal *R²* | LRT χ² (*df*) | *p* |
| --- | --- | --- | --- | --- | --- | --- | --- | --- | --- |
| Main-Effects | -306.00 | 620.00 | 637.48 | 612.00 | 580 | 0.32 | 0.01 | — | — |
| Interaction | -306.00 | 622.00 | 643.85 | 612.00 | 579 | 0.32 | 0.01 | 0.00 (1) | .988 |

Because the interaction did not contribute to better fit, the more parsimonious main-effects model was selected as the optimal model and is the focus of interpretation in the main text.

To explore age-related trends within children, we fitted another LMM on the child subsample (*N* = 388 observations from 97 participants). The model included fixed effects for age in years (continuous, mean-centered), modesty understanding (incorrect vs. correct), and their interaction (random intercept for participant ID). The full model did not provide a significantly better fit than the null model (Δ*LL* = 1.91, Δ*df* = 3, χ² = 3.82, *p* = .282), indicating limited evidence for age-related effects. Detailed fit indices for this comparison are presented in Table B1.16.

**Table B1.16**

*Model Fit Indices and Comparisons (Null vs. Full Model) – Learning Preferences (Children Only)*

| Model | LogLikelihood | AIC | BIC | Deviance | Residual *df* | Conditional *R²* | Marginal *R²* | LRT χ² (*df*) | *p* |
| --- | --- | --- | --- | --- | --- | --- | --- | --- | --- |
| Null | -198.06 | 400.13 | 408.05 | 396.13 | 386 | 0.15 | 0.00 | — | — |
| Full | -196.15 | 402.31 | 422.11 | 392.31 | 383 | 0.16 | 0.02 | 3.82 (3) | .282 |

***Praiseworthiness and Likability*** ***Judgments***

For each of the two judgments (i.e., praiseworthiness and likeability), we first ran a proportional test to examine participants’ overall preference between the protagonists, and then ran a binomial logistic regression to examine the potential differences between children and adults. First, 95% of all participants judged the truth-telling protagonist as more praiseworthy than the lie-telling protagonist, a rate significantly above chance, *p* < .001, 95% CI [.90, .98]. The binomial logistic regression results suggested that children were more likely to judge the truth-telling protagonist as more praiseworthy than the lie-teller compared to their adult counterpart, *B* = 2.59, *SE* = 1.10, *OR* = 13.40, 95% CI [1.56, 114.69], *p* = .018.

Second, 93% of all participants judged the truth-telling protagonist as more likable than the lie-telling protagonist, also a rate significantly above chance, *p* < .001, 95% CI [.88, .97]. The binomial logistic regression results indicated that children tended to judge the truth-telling protagonist as more likable than the lie-teller compared to their adult counterpart, *B* = 3.07, *SE* = 1.07, *OR* = 21.60, 95% CI [2.65, 176.15], *p* = .004.

Children and adults were asked to provide justifications for their judgments of the protagonists’ praiseworthiness and likeability. Two coders reviewed and coded the participants’ responses. After reviewing all the responses, we identified four common themes to explain participants’ judgments: (a) honesty-related explanations, (b) harmony-related explanations, (c) combined explanations of honesty and harmony, and (d) irrelevant or no response. The coding scheme is presented in Table B1.17.

The inter-rater reliabilities between the two coders were high. For coding the judgments on the praiseworthiness of the two protagonists, the intraclass correlation coefficient (ICC) agreement was 86%, indicating substantial agreement between the coders. The Light’s Kappa statistic was also high, at .83. For coding the judgments on the likeability of the two protagonists, the ICC agreement was also 86%, with a Light’s Kappa of 0.83. Any disagreements between the two coders were resolved after two rounds of discussion.

Table B1.17

*Coding Scheme of Participants’ Explanations of the Protagonists’ Behaviors*

| Theme | Definition | Example |
| --- | --- | --- |
| Honesty-Related | Descriptions of the protagonists’ thoughts, traits, or behaviors related to their intentions to deceive others or not. | “She is very honest.”  “He did not lie.” |
| Harmony-Related | Descriptions of the protagonists’ thoughts, traits, or behaviors related to their intentions to promote interpersonal relationships or not. | “Because she is very modest.”  “Because he is not proud, and he fit in [with the group] more quickly.” |
| Combined Honesty and Harmony | Descriptions that include both intentions to show honesty and to promote modesty as defined above. | “Because he is honest and loves other people.”  “Although the person with the square is modest, the person with the triangle is more honest when interacting with others.” |
| Irrelevant or No Response | Descriptions that are irrelevant to the questions or acknowledge that participants do not have concrete answers to the questions. | “[He is] running around the kindergarten.”  “I don’t know.” |

We analyzed the distribution of the four identified themes for each judgment item based on participants’ age groups. In terms of justifying their judgments of the protagonists’ praiseworthiness, the majority of participants mentioned the protagonists’ intentions to show honesty, with 90% of child participants and 82% of adult participants mentioning it (see Figure B1).

In terms of justifying their judgments of the protagonists’ likeability, the majority of participants still mentioned the protagonists’ intentions to show honesty, with 85% of child participants and 65% of adult participants mentioning it (see Figure B1). However, compared to children, adults also mentioned the intention to promote harmony more frequently, χ^2^(3, *N* = 146) = 15.49, *p* = .001.

**Figure B1**

*Frequencies of Child and Adult Participants’ Explanations of the Truth-Telling and Lie-Telling Protagonists’ Behaviors in Study 1A*


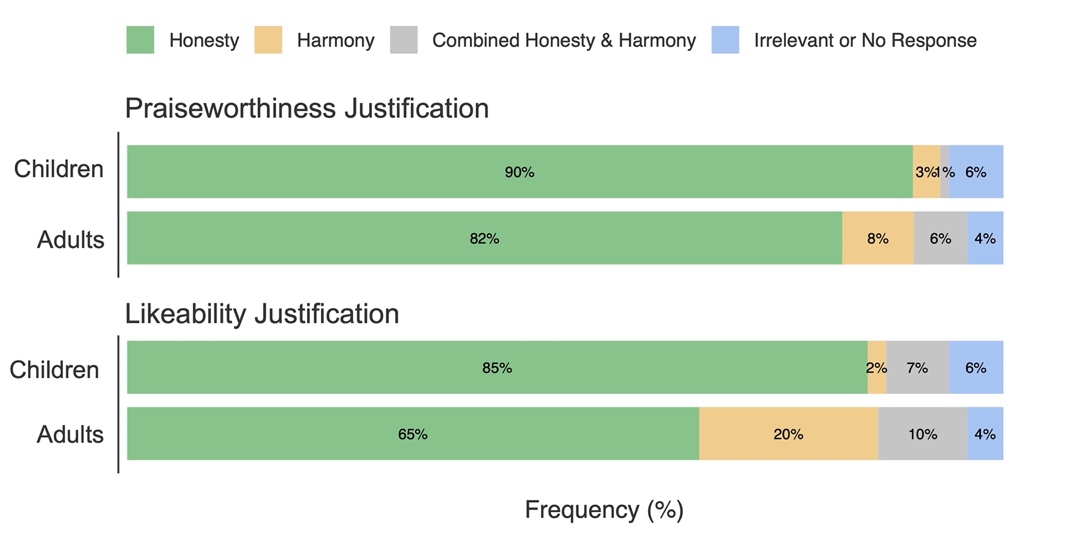


***Honesty and Modesty Understanding***

For each construct, participants completed two questions: an explicit self-report item (“Do you know the meaning of telling the truth [modesty]?”) and a forced-choice item identifying which protagonist did not tell the truth (for honesty) or which was more modest (for modesty). Correct responses on the forced-choice items were selecting the lie-telling protagonist for honesty understanding and the truth-telling protagonist for modesty understanding in Study 1A.

Regarding honesty, 100% of both child and adult participants (*N* = 146) reported understanding its meaning. Regarding modesty, all adult participants reported understanding the meaning of modesty. However, only 42% of child participants reported understanding its meaning, which did not differ significantly from chance (50%), *p* = .155. Children’s age was positively associated with their reported modesty understanding, *B* = 0.74, *OR* = 2.10, 95% CI [1.32, 3.44], *p* = .002. To provide a clearer picture of the development of modesty understanding, we report additional descriptive details for the child sample below. Children who correctly understood modesty were significantly older (*n* = 41; 16 girls and 25 boys; *M*_age_ = 8.69 years, *SD*_age_ = 1.13 years, age range = 6.44 to 11.33 years) than those who did not (*n* = 56; 31 girls and 25 boys; *M*_age_ = 7.99 years, *SD*_age_ = 0.85 years, age range = 5.79 to 10.54 years).

The age-specific patterns showed a clear developmental progression (see Table B1.18), with reported modesty understanding increasing steadily from age 6 years onward and reaching higher levels by ages 8 to 9 years. By contrast, adults exhibited ceiling-level explicit reports of modesty understanding (100%), substantially higher than the child rate of 42%. These patterns indicate that explicit awareness of modesty emerges gradually across middle to late childhood and is fully consolidated by adulthood.

**Table B1.18**

*Modesty Understanding by Age in Children (Study 1A)*

| Integer Age  (Years) | Reported  Modesty  Understanding | Counts | % of Total | Cumulative % |
| --- | --- | --- | --- | --- |
| 5 | No | 1 | 1% | 1% |
|  | Yes | 0 | 0% | 1% |
| 6 | No | 6 | 6% | 7% |
|  | Yes | 3 | 3% | 10% |
| 7 | No | 21 | 22% | 32% |
|  | Yes | 10 | 10% | 42% |
| 8 | No | 22 | 23% | 65% |
|  | Yes | 14 | 14% | 79% |
| 9 | No | 5 | 5% | 85% |
|  | Yes | 8 | 8% | 93% |
| 10 | No | 1 | 1% | 94% |
|  | Yes | 5 | 5% | 99% |
| 11 | No | 0 | 0% | 99% |
|  | Yes | 1 | 1% | 100% |

The explicit self-report items exhibited limited variance—particularly for honesty—and thus offered little discriminatory power. We therefore relied on the forced-choice protagonist selections as our primary measures of understanding, as they more directly reflected participants’ context-specific comprehension of the constructs.

Across the full sample *(N* = 146), forced-choice accuracy was 95% for identifying the dishonest protagonist (honesty understanding) and 90% for identifying the modest protagonist (modesty understanding).

Regarding honesty understanding (forced-choice item), children performed near ceiling, with 99% correct (96/97) compared to 88% correct among adults (43/49). A chi-square test of independence revealed a significant age-group difference, Pearson χ²(1, *N* = 146) = 8.97, *p* = .003. Given low expected frequencies in some cells (< 5), Yates’ continuity correction yielded χ²(1) = 6.68, *p* = .010, confirming that children outperformed adults.

Regarding modesty understanding, explicit self-reports showed a marked developmental difference—all adults (100%) reported knowing the meaning of modesty, whereas only 42% of children did so. However, participants’ accuracy in answering the forced-choice question was high in both groups: 88% of children (85/97) and 96% of adults (47/49) correctly identified the modest protagonist. A chi-square test showed no significant age-group difference, Pearson χ²(1, *N* = 146) = 2.58, *p* = .108 (Yates’ corrected χ²(1) = 1.71, *p* = .191). This pattern indicates that while explicit awareness of modesty lags in childhood, context-specific (implicit) comprehension is already strong in most children.

Among children only, we tested whether continuous age in years predicted accuracy (forced-choice accuracy) using separate logistic regressions. Age did not significantly predict accuracy on honesty understanding (*B* = 0.47, *SE* = 0.32, *OR* = 1.59, 95% CI [0.85, 2.98], *p* = .144) or on modesty understanding (*B* = 0.32, *SE* = 0.21, *OR* = 1.38, 95% CI [0.91, 2.08], *p* = .128). Although accuracy tended to increase slightly with age, these associations did not reach statistical significance.

**Study 1B**

**Warmth Evaluation Task**

In the Warmth Evaluation Task, participants’ choices of the truth-telling protagonist (coded as 1) versus the lie-telling protagonist (coded as 0) were analyzed using generalized linear mixed models (LMMs) with a binomial distribution and logit link. All models included a random intercept for participant ID to account for the clustering of responses across items.

We fitted three nested models. First, we estimated a null (intercept-only) model, which captured the overall marginal preference for the truth-telling protagonist across the sample (reported in the main text). Next, we added fixed main effects for age group (children vs. adults), honesty understanding (incorrect vs. correct), and modesty understanding (incorrect vs. correct). All predictors were deviation-coded (simple coding), ensuring that the model intercept represented the predicted log-odds of preferring the truth-telling protagonist at the average level of each predictor. This main-effects model is also reported in the main text.

A likelihood-ratio test showed that the main-effects model provided a significantly better fit than the null model (Δ*LL* = 42.50, Δ*df* = 3, χ² = 84.99, *p* < .001), indicating that the three predictors collectively explained meaningful variance in preferences. Detailed fit indices for these models are presented in Table B2.1.

**Table B2.1**

*Model Fit Indices and Comparisons (Null vs. Main-Effects Model) for Warmth Evaluations*

| Model | LogLikelihood | AIC | BIC | Deviance | Residual *df* | Conditional *R²* | Marginal *R²* | LRT χ² (*df*) | *p* |
| --- | --- | --- | --- | --- | --- | --- | --- | --- | --- |
| Null | -338.95 | 681.91 | 690.65 | 677.91 | 582 | 0.59 | 0.00 | — | — |
| Main-Effects | -296.46 | 602.92 | 624.77 | 592.92 | 579 | 0.60 | 0.37 | 84.99 (3) | < .001 |

Next, we tested a more complex interaction model that retained the main effects and added the two-way interactions involving age group: Specifically, the interaction between age group and honesty understanding, and the interaction between age group and modesty understanding. Parameter estimates for this model are provided in Table B2.2.

**Table B2.2**

*Fixed-Effects Parameter Estimates for the Interaction Model for Warmth Evaluations*

|  | Estimate *(B)* | *SE* | *OR* | 95% CI  Lower | 95% CI  Upper | *z* | *p* |
| --- | --- | --- | --- | --- | --- | --- | --- |
| Intercept | 0.36 | 0.34 | 1.43 | 0.74 | 2.78 | 1.07 | .286 |
| Age Group | -0.13 | 0.67 | 0.88 | 0.24 | 3.29 | -0.19 | .850 |
| Honesty Understanding | 2.83 | 0.59 | 16.92 | 5.32 | 53.82 | 4.79 | < .001 |
| Modesty Understanding | -3.49 | 0.72 | 0.03 | 0.01 | 0.13 | -4.82 | < .001 |
| Age Group × Honesty Understanding | 1.44 | 1.14 | 4.23 | 0.45 | 39.57 | 1.27 | .206 |
| Age Group × Modesty Understanding | -1.21 | 1.39 | 0.30 | 0.02 | 4.58 | -0.87 | .386 |

 A likelihood-ratio test indicated that adding the two-way interactions did not significantly improve model fit over the main-effects model (Δ*LL* 1.01, Δ*df* = 2, χ² = 2.02, *p* = .364). Detailed fit indices for this comparison are presented in Table B2.3.

**Table B2.3**

*Model Fit Indices and Comparisons (Main-Effects vs. Interaction Model) for Warmth Evaluations*

| Model | LogLikelihood | AIC | BIC | Deviance | Residual *df* | Conditional *R²* | Marginal *R²* | LRT χ² (*df*) | *p* |
| --- | --- | --- | --- | --- | --- | --- | --- | --- | --- |
| Main-Effects | -296.46 | 602.92 | 624.77 | 592.92 | 579 | 0.60 | 0.37 | — | — |
| Interaction | -295.45 | 604.89 | 635.48 | 590.89 | 577 | 0.61 | 0.39 | 2.02 (2) | .364 |

Because the interactions did not contribute to better fit, the more parsimonious main-effects model was selected as the optimal model and is the focus of interpretation in the main text.

To explore age-related trends within children, we fitted another LMM on the child subsample (*N* = 388 observations from 97 participants). Models included fixed effects for age in years (continuous, mean-centered), honesty understanding (correct vs. incorrect), modesty understanding (correct vs. incorrect), and the two-way interactions between age and each understanding variable. All models included a random intercept for participant ID. Below, we report model fit comparisons with the null (intercept-only) model for warmth evaluations task. The full model provided a significantly better fit than the null model (*ΔLL* = 34.31, *Δdf* = 5, χ² = 68.62, *p* < .001), indicating that the predictors collectively explained meaningful variance. Detailed fit indices for these models are presented in Table B2.4.

**Table B2.4**

*Model Fit Indices and Comparisons (Null vs. Full Model) – Warmth Evaluations (Children Only)*

| Model | LogLikelihood | AIC | BIC | Deviance | Residual *df* | Conditional *R²* | Marginal *R²* | LRT χ² (*df*) | *p* |
| --- | --- | --- | --- | --- | --- | --- | --- | --- | --- |
| Null | -215.66 | 435.33 | 443.25 | 431.33 | 386 | 0.61 | 0.00 | — | — |
| Full | -181.35 | 376.71 | 404.43 | 362.71 | 381 | 0.59 | 0.41 | 68.62 (5) | < .001 |

**Competence Evaluation Task**

In the Competence Evaluation Task, participants’ choices of the truth-telling protagonist (coded as 1) versus the lie-telling protagonist (coded as 0) were analyzed using LMMs with a binomial distribution and logit link. All models included a random intercept for participant ID to account for the clustering of responses across items.

We fitted three nested models. First, we estimated a null (intercept-only) model, which captured the overall marginal preference for the truth-telling protagonist across the sample (reported in the main text). Next, we added fixed main effects for age group, honesty understanding, and modesty understanding. All predictors were deviation-coded (simple coding). This main-effects model is also reported in the main text.

A likelihood-ratio test showed that the main-effects model provided a significantly better fit than the null model (Δ*LL* = 26.03, Δ*df* = 3, χ² = 52.05, *p* < .001), indicating that the three predictors collectively explained meaningful variance in preferences. Detailed fit indices for these models are presented in Table B2.5.

**Table B2.5**

*Model Fit Indices and Comparisons (Null vs. Main-Effects Model) for Competence Evaluations*

| Model | LogLikelihood | AIC | BIC | Deviance | Residual *df* | Conditional *R²* | Marginal *R²* | LRT χ² (*df*) | *p* |
| --- | --- | --- | --- | --- | --- | --- | --- | --- | --- |
| Null | -315.22 | 634.43 | 643.17 | 630.43 | 582 | 0.70 | 0.00 | — | — |
| Main-Effects | -289.19 | 588.38 | 610.23 | 578.38 | 579 | 0.72 | 0.28 | 52.05 (3) | < .001 |

Then, we tested a more complex interaction model that retained the main effects and added the two-way interactions involving age group: Specifically, the interaction between age group and honesty understanding, and the interaction between age group and modesty understanding. Parameter estimates for this model are provided in Table B2.6.

**Table B2.6**

*Fixed-Effects Parameter Estimates for the Interaction Model for Competence Evaluations*

|  | Estimate *(B)* | *SE* | *OR* | 95% CI  Lower | 95% CI  Upper | *z* | *p* |
| --- | --- | --- | --- | --- | --- | --- | --- |
| Intercept | 0.90 | 0.45 | 2.46 | 1.01 | 6.00 | 1.98 | .048 |
| Age Group | 0.59 | 0.90 | 1.80 | 0.31 | 10.40 | 0.66 | .512 |
| Honesty Understanding | 2.11 | 0.72 | 8.22 | 2.01 | 33.63 | 2.93 | .003 |
| Modesty Understanding | -3.77 | 0.92 | 0.02 | 0.00 | 0.14 | -4.09 | < .001 |
| Age Group × Honesty Understanding | -0.63 | 1.39 | 0.53 | 0.04 | 8.11 | -0.45 | .651 |
| Age Group × Modesty Understanding | -1.40 | 1.72 | 0.25 | 0.01 | 7.22 | -0.81 | .417 |

A likelihood-ratio test indicated that adding the two-way interactions did not significantly improve model fit over the main-effects model (Δ*LL* = 0.53, Δ*df* = 2, χ² = 1.06, *p* = .588). Detailed fit indices for this comparison are presented in Table B2.7.

**Table B2.7**

*Model Fit Indices and Comparisons (Main-Effects vs. Interaction Model) for Competence Evaluations*

| Model | LogLikelihood | AIC | BIC | Deviance | Residual *df* | Conditional *R²* | Marginal *R²* | LRT χ² (*df*) | *p* |
| --- | --- | --- | --- | --- | --- | --- | --- | --- | --- |
| Main-Effects | -289.19 | 588.38 | 610.23 | 578.38 | 579 | 0.72 | 0.28 | — | — |
| Interaction | -288.66 | 591.32 | 621.91 | 577.32 | 577 | 0.72 | 0.29 | 1.06 (2) | .588 |

Because the interactions did not contribute to better fit, the more parsimonious main-effects model was selected as the optimal model and is the focus of interpretation in the main text.

To explore age-related trends within children, we fitted another LMM on the child subsample (*N* = 388 observations from 97 participants). Models included fixed effects for age in years (continuous, mean-centered), honesty understanding (correct vs. incorrect), modesty understanding (correct vs. incorrect), and the two-way interactions between age and each understanding variable. All models included a random intercept for participant ID. Below, we report model fit comparisons with the null (intercept-only) model for competence evaluations task. The full model provided a significantly better fit than the null model (*ΔLL* = 24.80, *Δdf* = 5, χ² = 49.61, *p* < .001), indicating that the predictors collectively explained meaningful variance. Detailed fit indices for these models are presented in Table B2.8.

**Table B2.8**

*Model Fit Indices and Comparisons (Null vs. Full Model) – Competence Evaluations (Children Only)*

| Model | LogLikelihood | AIC | BIC | Deviance | Residual *df* | Conditional *R²* | Marginal *R²* | LRT χ² (*df*) | *p* |
| --- | --- | --- | --- | --- | --- | --- | --- | --- | --- |
| Null | -210.92 | 425.84 | 433.76 | 421.84 | 386 | 0.64 | 0.00 | — | — |
| Full | -186.12 | 386.24 | 413.96 | 372.24 | 381 | 0.64 | 0.34 | 49.61 (5) | < .001 |

**Socializing Preferences Task**

In the Socializing Preferences Task, participants’ choices of the truth-telling protagonist (coded as 1) versus the lie-telling protagonist (coded as 0) were analyzed using LMMs with a binomial distribution and logit link. All models included a random intercept for participant ID to account for the clustering of responses across items.

We fitted three nested models. First, we estimated a null (intercept-only) model, which captured the overall marginal preference for the truth-telling protagonist across the sample (reported in the main text). Next, we added fixed main effects for age group, honesty understanding, and modesty understanding. All predictors were deviation-coded (simple coding). This main-effects model is also reported in the main text.

A likelihood-ratio test showed that the main-effects model provided a significantly better fit than the null model (Δ*LL* = 33.56, Δ*df* = 3, χ² = 67.12, *p* < .001), indicating that the three predictors collectively explained meaningful variance in preferences. Detailed fit indices for these models are presented in Table B2.9.

**Table B2.9**

*Model Fit Indices and Comparisons (Null vs. Main-Effects Model) for Socializing Preferences*

| Model | LogLikelihood | AIC | BIC | Deviance | Residual *df* | Conditional *R²* | Marginal *R²* | LRT χ² (*df*) | *p* |
| --- | --- | --- | --- | --- | --- | --- | --- | --- | --- |
| Null | -267.59 | 539.19 | 547.93 | 535.19 | 582 | 0.84 | 0.00 | — | — |
| Main-Effects | -234.03 | 478.07 | 499.92 | 468.07 | 579 | 0.86 | 0.37 | 67.12 (3) | < .001 |

Next, we tested a more complex interaction model that retained the main effects and added the two-way interactions involving age group: Specifically, the interaction between age group and honesty understanding, and the interaction between age group and modesty understanding. Parameter estimates for this model are provided in Table B2.10.

**Table B2.10**

*Fixed-Effects Parameter Estimates for the Interaction Model for Socializing Preferences*

|  | Estimate *(B)* | *SE* | *OR* | 95% CI  Lower | 95% CI  Upper | *z* | *p* |
| --- | --- | --- | --- | --- | --- | --- | --- |
| Intercept | 1.31 | 0.64 | 3.71 | 1.06 | 13.01 | 2.04 | .041 |
| Age Group | 0.73 | 1.27 | 2.07 | 0.17 | 24.86 | 0.57 | .566 |
| Honesty Understanding | 3.83 | 1.07 | 46.05 | 5.66 | 374.78 | 3.58 | < .001 |
| Modesty Understanding | -4.83 | 1.25 | 0.01 | 0.00 | 0.09 | -3.86 | < .001 |
| Age Group × Honesty Understanding | -0.33 | 1.95 | 0.72 | 0.02 | 32.58 | -0.17 | .865 |
| Age Group × Modesty Understanding | 1.30 | 2.34 | 3.69 | 0.04 | 359.38 | 0.56 | .577 |

A likelihood-ratio test indicated that adding the two-way interactions did not significantly improve model fit over the main-effects model (Δ*LL* = 0.15, Δ*df* = 2, χ² = 0.30, *p* = .862). Detailed fit indices for this comparison are presented in Table B2.11.

**Table B2.11**

*Model Fit Indices and Comparisons (Main-Effects vs. Interaction Model)* *for Socializing Preferences*

| Model | LogLikelihood | AIC | BIC | Deviance | Residual *df* | Conditional *R²* | Marginal *R²* | LRT χ² (*df*) | *p* |
| --- | --- | --- | --- | --- | --- | --- | --- | --- | --- |
| Main-Effects | -234.03 | 478.07 | 499.92 | 468.07 | 579 | 0.86 | 0.37 | — | — |
| Interaction | -233.89 | 481.77 | 512.36 | 467.77 | 577 | 0.86 | 0.37 | 0.30 (2) | .862 |

Because the interactions did not contribute to better fit, the more parsimonious main-effects model was selected as the optimal model and is the focus of interpretation in the main text.

To explore age-related trends within children, we fitted another LMM on the child subsample (*N* = 388 observations from 97 participants). Models included fixed effects for age in years (continuous, mean-centered), honesty understanding (correct vs. incorrect), modesty understanding (correct vs. incorrect), and the two-way interactions between age and each understanding variable. All models included a random intercept for participant ID. Below, we report model fit comparisons with the null (intercept-only) model for socializing preferences task. The full model provided a significantly better fit than the null model (*ΔLL* = 33.16, *Δdf* = 5, χ² = 66.31, *p* < .001), indicating that the predictors collectively explained meaningful variance. Detailed fit indices for these models are presented in Table B2.12.

**Table B2.12**

*Model Fit Indices and Comparisons (Null vs. Full Model) – Socializing Preferences (Children Only)*

| Model | LogLikelihood | AIC | BIC | Deviance | Residual *df* | Conditional *R²* | Marginal *R²* | LRT χ² (*df*) | *p* |
| --- | --- | --- | --- | --- | --- | --- | --- | --- | --- |
| Null | -175.19 | 354.37 | 362.29 | 350.37 | 386 | 0.85 | 0.00 | — | — |
| Full | -142.03 | 298.06 | 325.79 | 284.06 | 381 | 0.85 | 0.51 | 66.31 (5) | < .001 |

**Learning Preferences Task**

In the Learning Preferences Task, participants’ choices of the truth-telling protagonist (coded as 1) versus the lie-telling protagonist (coded as 0) were analyzed using LMMs with a binomial distribution and logit link. All models included a random intercept for participant ID to account for the clustering of responses across items.

We fitted three nested models. First, we estimated a null (intercept-only) model, which captured the overall marginal preference for the truth-telling protagonist across the sample (reported in the main text). Next, we added fixed main effects for age group, honesty understanding, and modesty understanding. All predictors were deviation-coded (simple coding). This main-effects model is also reported in the main text.

A likelihood-ratio test showed that the main-effects model provided a significantly better fit than the null model (Δ*LL* = 9.16, Δ*df* = 3, χ² = 18.33, *p* < .001), indicating that the three predictors collectively explained meaningful variance in preferences. Detailed fit indices for these models are presented in Table B2.13.

**Table B2.13**

*Model Fit Indices and Comparisons (Null vs. Main-Effects Model) for Learning Preferences*

| Model | LogLikelihood | AIC | BIC | Deviance | Residual *df* | Conditional *R²* | Marginal *R²* | LRT χ² (*df*) | *p* |
| --- | --- | --- | --- | --- | --- | --- | --- | --- | --- |
| Null | -333.81 | 671.62 | 680.36 | 667.62 | 582 | 0.57 | 0.00 | — | — |
| Main-Effects | -324.65 | 659.29 | 681.14 | 649.29 | 579 | 0.59 | 0.10 | 18.33 (3) | < .001 |

Next, we tested a more complex interaction model that retained the main effects and added the two-way interactions involving age group: Specifically, the interaction between age group and honesty understanding, and the interaction between age group and modesty understanding. Parameter estimates for this model are provided in Table B2.14.

**Table B2.14**

*Fixed-Effects Parameter Estimates for the Interaction Model for Learning Preferences*

|  | Estimate *(B)* | *SE* | *OR* | 95% CI  Lower | 95% CI  Upper | *z* | *p* |
| --- | --- | --- | --- | --- | --- | --- | --- |
| Intercept | 1.44 | 0.43 | 4.21 | 1.82 | 9.77 | 3.35 | < .001 |
| Age Group | 0.86 | 0.83 | 2.36 | 0.46 | 12.03 | 1.04 | .300 |
| Honesty Understanding | 1.00 | 0.61 | 2.71 | 0.83 | 8.89 | 1.65 | .100 |
| Modesty Understanding | -2.67 | 0.84 | 0.07 | 0.01 | 0.36 | -3.18 | .001 |
| Age Group × Honesty Understanding | 1.79 | 1.21 | 5.98 | 0.56 | 64.33 | 1.47 | .140 |
| Age Group × Modesty Understanding | -1.64 | 1.63 | 0.19 | 0.01 | 4.67 | -1.01 | .312 |

A likelihood-ratio test indicated that adding the two-way interactions did not significantly improve model fit over the main-effects model (Δ*LL* = 1.48, Δ*df* = 2, χ² = 2.96, *p* = .228). Detailed fit indices for this comparison are presented in Table B2.15.

**Table B2.15**

*Model Fit Indices and Comparisons (Main-Effects vs. Interaction Model) for Learning Preferences*

| Model | LogLikelihood | AIC | BIC | Deviance | Residual *df* | Conditional *R²* | Marginal *R²* | LRT χ² (*df*) | *p* |
| --- | --- | --- | --- | --- | --- | --- | --- | --- | --- |
| Main-Effects | -324.65 | 659.29 | 681.14 | 649.29 | 579 | 0.59 | 0.10 | — | — |
| Interaction | -323.17 | 660.34 | 690.92 | 646.34 | 577 | 0.60 | 0.13 | 2.96 (2) | .228 |

Because the interactions did not contribute to better fit, the more parsimonious main-effects model was selected as the optimal model and is the focus of interpretation in the main text.

To explore age-related trends within children, we fitted another LMM on the child subsample (*N* = 388 observations from 97 participants). Models included fixed effects for age in years (continuous, mean-centered), honesty understanding (correct vs. incorrect), modesty understanding (correct vs. incorrect), and the two-way interactions between age and each understanding variable. All models included a random intercept for participant ID. Below, we report model fit comparisons with the null (intercept-only) model for learning preferences task. The full model provided a significantly better fit than the null model (*ΔLL* = 9.45, *Δdf* = 5, χ² = 18.89, *p* = .002), indicating that the predictors collectively explained meaningful variance. Detailed fit indices for this comparison are presented in Table B2.16.

**Table B2.16**

*Model Fit Indices and Comparisons (Null vs. Full Model) – Learning Preferences (Children Only)*

| Model | LogLikelihood | AIC | BIC | Deviance | Residual *df* | Conditional *R²* | Marginal *R²* | LRT χ² (*df*) | *p* |
| --- | --- | --- | --- | --- | --- | --- | --- | --- | --- |
| Null | -232.52 | 469.04 | 476.96 | 465.04 | 386 | 0.47 | 0.00 | — | — |
| Full | -223.07 | 460.14 | 487.87 | 446.14 | 381 | 0.49 | 0.13 | 18.89 (5) | .002 |

***Praiseworthiness and Likability*** ***Judgments***

For each of the two judgments (i.e., praiseworthiness and likeability), we first ran a proportional test to examine participants’ overall preference between the protagonists, then ran a binomial logistic regression to examine the potential differences between children and adults. First, 63% of all participants judged the truth-telling protagonist as more praiseworthy than the lie-telling protagonist, which significantly differed from chance, *p* = .002, 95% CI [.55, .71]. The binomial logistic regression results suggested that children were more likely to judge the truth-telling protagonist as more praiseworthy than the lie-telling protagonist compared to their adult counterpart, *B* = 0.76, *SE* = 0.36, *OR* = 2.14, 95% CI [1.06, 4.35], *p* = .034.

In terms of the protagonists’ likeability, 55% of all participants judged the truth-telling protagonist as more likable than the lie-telling protagonist, which did not significantly differ from chance, *p* = .282, 95% CI [.46, .63]. The binomial logistic regression results indicated that children tended to judge the truth-telling protagonist as more likable than the lie-telling protagonist compared to their adult counterpart, *B* = 1.12, *SE* = 0.36, *OR* = 3.05, 95% CI [1.49, 6.23], *p* = .002.

Children and adults were asked to provide justifications for their judgments of the protagonists’ praiseworthiness and likeability. As with Study 1A, two coders reviewed and coded the participants’ responses. The identified common themes were the same with Study 1A (see Table B1.17): (a) honesty-related explanations, (b) harmony-related explanations, (c) combined explanations of honesty and harmony, and (d) irrelevant or no response.

The inter-rater reliabilities between the two coders were high. For coding the judgments on the praiseworthiness of the two protagonists, the ICC agreement was 89%, indicating substantial agreement between the coders. The Light’s Kappa statistic was also high, at .91. For coding the judgments on the likeability of the two protagonists, the ICC agreement was also 88%, with a Light’s Kappa of .93. Any disagreements between the two coders were resolved after two rounds of discussion.

We next examined the distribution of the four identified justification themes as a function of age group. For judgments of the protagonists’ praiseworthiness, most participants referenced the protagonists’ intentions to be honest when explaining their judgments; this was especially pronounced among children, with 71% of child participants mentioning honesty-related intentions, compared with 49% of adult participants (see Figure B2). By contrast, harmony-related explanations were less frequently used by children (21%) than by adults (41%).

**Figure B2**

*Frequencies of Child and Adult Participants’ Explanations of the Truth-Telling and Lie-Telling Protagonists’ Behaviors in Study 1B*


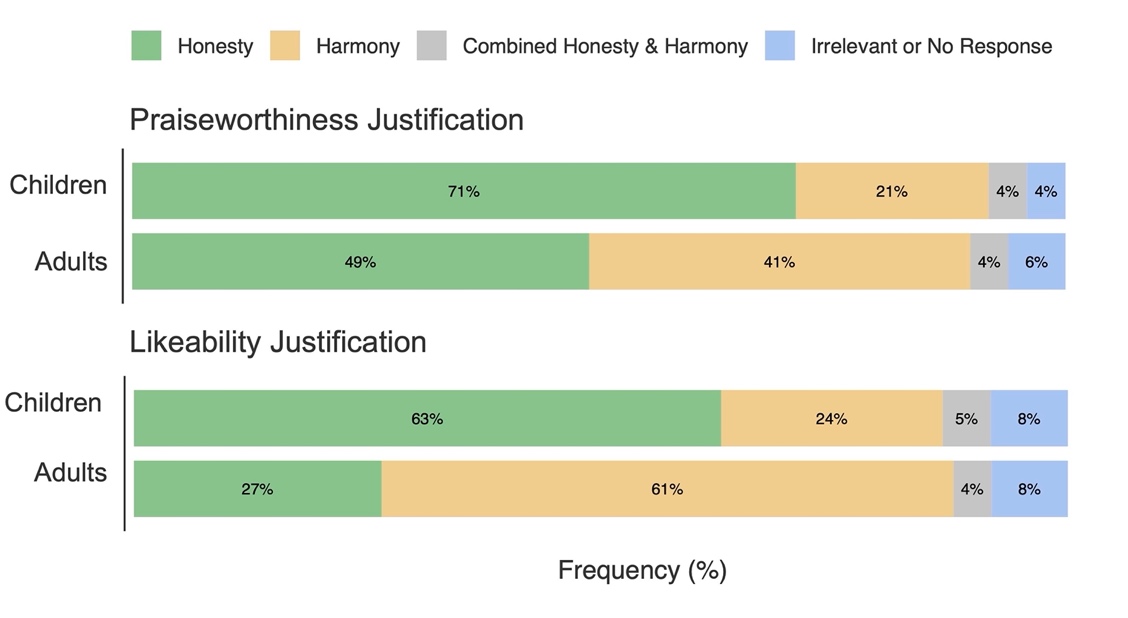


Age-related differences were also evident in justifications of likeability judgments (see Figure B2). A majority of children emphasized honesty-related intentions (63%), whereas fewer children referenced the promotion of harmony (24%). Adults showed the opposite pattern: most adults justified their likeability judgments in terms of harmony-related intentions (61%), with a smaller proportion referencing honesty-related intentions (27%). Overall, these findings indicate that children tend to prioritize honesty over harmony when explaining their likeability judgments, whereas adults place greater weight on the promotion of social harmony than on honesty.

***Honesty and Modesty Understanding***

For each construct, participants completed two questions: an explicit self-report item (“Do you know the meaning of telling the truth [modesty]?”) and a forced-choice item identifying which protagonist did not tell the truth (for honesty) or which was more modest (for modesty). Correct responses on the forced-choice items were selecting the lie-telling protagonist for honesty understanding and modesty understanding in Study 1B. We relied on the forced-choice protagonist selections as our primary measures of understanding, as they more directly reflected participants’ context-specific comprehension of the constructs.

Across the full sample *(N* = 146), forced-choice accuracy was 84% for identifying the dishonest protagonist (honesty understanding) and 67% for identifying the modest protagonist (modesty understanding).

Regarding honesty understanding (forced-choice item), 86% of children (83/97) answered correctly compared to 82% of adults (40/49). A chi-square test of independence revealed no significant age-group difference, Pearson χ²(1, *N* = 146) = 0.38, *p* = .538 ( Yates’ corrected χ²(1) = 0.14, *p* = .707.

Regarding modesty understanding, only 56% of children (54/97) answered correctly compared to 90% of adults (44/49). A chi-square test revealed a significant age-group difference, Pearson χ²(1, *N* = 146) = 17.18, *p* < .001 (Yates’ corrected χ²(1) = 15.67, *p* < .001).

This pattern indicates a clear developmental progression in context-specific modesty understanding, with adults showing substantially better comprehension than children. By contrast, honesty understanding was high and comparable across age groups.

Among children only, we tested whether continuous age in years predicted comprehension accuracy using separate logistic regressions. Age did not significantly predict accuracy on honesty understanding (*B* = 0.47, *p* = .144, *OR* = 1.59, 95% CI [0.85, 2.98]) or on modesty understanding (*B* = 0.32, *p* = .128, *OR* = 1.38, 95% CI [0.91, 2.08]). Although accuracy tended to increase slightly with age, these associations did not reach statistical significance.

**Study 2**

To examine differences between Study 1B and Study 2 while controlling for individual differences in honesty and modesty understanding, we combined data from both studies (total *N* = 1076 observations from 269 participants) and fitted LMMs with a binomial distribution and logit link for each evaluation task separately. All models included a random intercept for participant ID. Predictors (Study, age group, honesty understanding, modesty understanding) were deviation-coded (simple coding).

Below, we report model fit comparisons with the null (intercept-only) model for each task to demonstrate the overall contribution of the predictors.

**Warmth Evaluation Task**

The main-effects model included fixed effects for Study (Study 1b vs. Study 2), age group, honesty understanding, and modesty understanding. This model provided a significantly better fit than the null model (Δ*LL* = 52.23, Δ*df* = 4, χ² = 104.46, *p* < .001), indicating that the predictors collectively explained meaningful variance (see Table B3.1).

**Table B3.1**

*Model Fit Indices and Comparisons (Null vs. Main-Effects Model) for Warmth Evaluations*

| Model | LogLikelihood | AIC | BIC | Deviance | Residual *df* | Conditional *R²* | Marginal *R²* | LRT χ² (*df*) | *p* |
| --- | --- | --- | --- | --- | --- | --- | --- | --- | --- |
| Null | -625.94 | 1255.87 | 1265.83 | 1251.87 | 1074 | 0.58 | 0.00 | — | — |
| Main-Effects | -573.70 | 1159.41 | 1189.29 | 1147.41 | 1070 | 0.61 | 0.27 | 104.46 (4) | < .001 |

**Competence Evaluation Task**

The main-effects model included fixed effects for Study (Study 1b vs. Study 2), age group, honesty understanding, and modesty understanding. This model provided a significantly better fit than the null model (Δ*LL* = 26.40, Δ*df* = 4, χ² = 52.80, *p* < .001), indicating that the predictors collectively explained meaningful variance (see Table B3.2).

**Table B3.2**

*Model Fit Indices and Comparisons (Null vs. Main-Effects Model) for Competence Evaluations*

| Model | LogLikelihood | AIC | BIC | Deviance | Residual *df* | Conditional *R²* | Marginal *R²* | LRT χ² (*df*) | *p* |
| --- | --- | --- | --- | --- | --- | --- | --- | --- | --- |
| Null | -613.86 | 1231.72 | 1241.68 | 1227.72 | 1074 | 0.64 | 0.00 | — | — |
| Main-Effects | -587.46 | 1186.92 | 1216.81 | 1174.92 | 1070 | 0.65 | 0.16 | 52.80 (4) | < .001 |

**Socializing Preferences Task**

The main-effects model included fixed effects for Study (Study 1b vs. Study 2), age group, honesty understanding, and modesty understanding. This model provided a significantly better fit than the null model (Δ*LL* = 47.30, Δ*df* = 4, χ² = 94.60, *p* < .001), indicating that the predictors collectively explained meaningful variance (see Table B3.3).

**Table B3.3**

*Model Fit Indices and Comparisons (Null vs. Main-Effects Model) for Socializing Preferences*

| Model | LogLikelihood | AIC | BIC | Deviance | Residual *df* | Conditional *R²* | Marginal *R²* | LRT χ² (*df*) | *p* |
| --- | --- | --- | --- | --- | --- | --- | --- | --- | --- |
| Null | -548.93 | 1101.85 | 1111.82 | 1097.85 | 1074 | 0.75 | 0.00 | — | — |
| Main-Effects | -501.63 | 1015.26 | 1045.14 | 1003.26 | 1070 | 0.77 | 0.29 | 94.60 (4) | < .001 |

**Learning Preferences Task**

The main-effects model included fixed effects for Study (Study 1b vs. Study 2), age group, honesty understanding, and modesty understanding. This model provided a significantly better fit than the null model (Δ*LL* = 11.99, Δ*df* = 4, χ² = 23.98, *p* < .001), indicating that the predictors collectively explained meaningful variance (see Table B3.4).

**Table B3.4**

*Model Fit Indices and Comparisons (Null vs. Main-Effects Model) for Learning Preferences*

| Model | LogLikelihood | AIC | BIC | Deviance | Residual *df* | Conditional *R²* | Marginal *R²* | LRT χ² (*df*) | *p* |
| --- | --- | --- | --- | --- | --- | --- | --- | --- | --- |
| Null | -661.46 | 1326.93 | 1336.89 | 1322.93 | 1074 | 0.48 | 0.00 | — | — |
| Main-Effects | -649.47 | 1310.95 | 1340.84 | 1298.95 | 1070 | 0.49 | 0.07 | 23.98 (4) | < .001 |

***Praiseworthiness and Likability*** ***Judgments***

For each of the two judgments (i.e., praiseworthiness and likeability), we first ran a proportional test to examine participants’ overall preference between the protagonists, then ran a binomial logistic regression to examine the potential differences between children and adults. First, 60% of all participants judged the truth-telling protagonist as more praiseworthy than the lie-telling protagonist, which significantly differed from chance, *p* = .031, 95% CI [.51, .69]. The binomial logistic regression results suggested no significant effect of age group (children vs. adults) on judging the truth-telling protagonist as more praiseworthy, *B* = 0.23, *SE* = 0.38, *OR* = 1.25, 95% CI [0.60, 2.62], *p* = .550.

In terms of the protagonists’ likeability, 74% of all participants judged the truth-telling protagonist as more likable than the lie-telling protagonist, which slightly differed from chance, *p* = .049, 95% CI [.50, .68]. The binomial logistic regression results indicated no significant difference between children and adults in their likelihood of judging the truth-teller as more likable, *B* = -.00, *SE* = 0.37, *OR* = 1.00, 95% CI [0.48, 2.07], *p* = .998.

Children and adults were asked to provide justifications for their judgments of the protagonists’ praiseworthiness and likeability. As with Study 1, two coders reviewed and coded the participants’ responses. The identified common themes were the same with Study 1 (see Table B1.17): (a) honesty-related explanations, (b) harmony-related explanations, (c) combined explanations of honesty and harmony, and (d) irrelevant or no response.

The inter-rater reliabilities between the two coders were high. For coding the judgments on the praiseworthiness of the two protagonists, the ICC agreement was 92%, indicating substantial agreement between the coders. The Light’s Kappa statistic was also high, at .93. For coding the judgments on the likeability of the two protagonists, the ICC agreement was also 89%, with a Light’s Kappa of .91. Any disagreements between the two coders were resolved after two rounds of discussion.

We next examined the distribution of these justification themes as a function of age group. For judgments of praiseworthiness, clear age-related differences emerged (see Figure B3). Children were less likely than adults to reference honesty-related intentions (22% vs. 41%) and harmony-related intentions (20% vs. 35%) when explaining their judgments.

A similar developmental pattern was observed for justifications of likeability judgments (see Figure B3). Among children, 22% referred to honesty-related intentions and 21% referenced the promotion of harmony. By contrast, adults predominantly justified their likeability judgments in terms of harmony-related considerations (45%), with a smaller proportion referencing honesty-related intentions (29%).

**Figure B3**

*Frequencies of Child and Adult Participants’ Explanations of the Truth-Telling and Lie-Telling Protagonists’ Behaviors in Study 2*


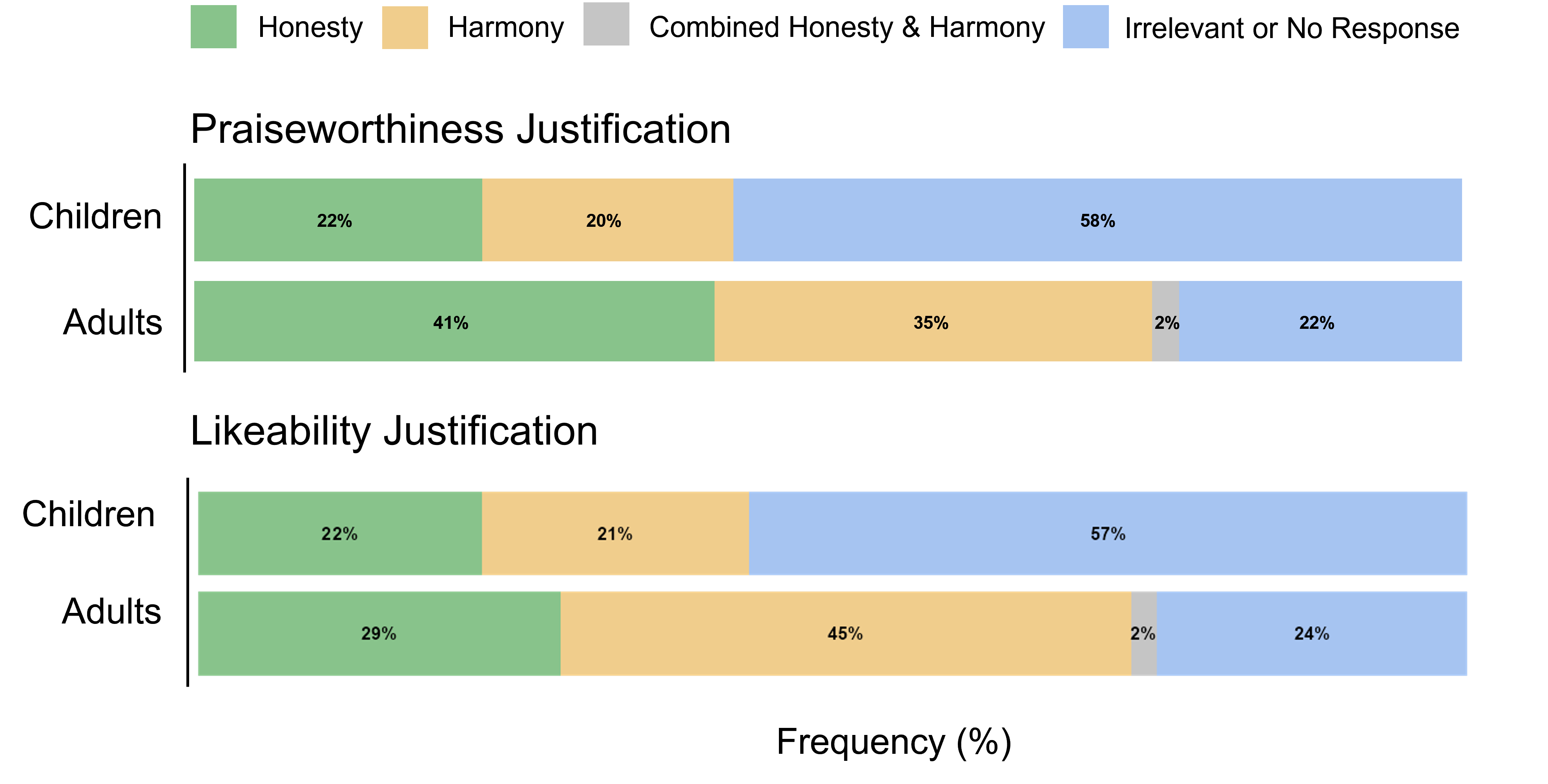


***Honesty and Modesty Understanding***

For each construct, participants completed two questions: an explicit self-report item (“Do you know the meaning of telling the truth [modesty]?”) and a forced-choice item identifying which protagonist did not tell the truth (for honesty) or which was more modest (for modesty). Correct responses on the forced-choice items were selecting the lie-telling protagonist for honesty understanding and modesty understanding in Study 2.

Regarding honesty, 100% of both child and adult participants (*N* = 125) reported understanding its meaning. Regarding modesty, 98% of adult participants (*n* = 48; 24 women and 24 men) reported understanding the meaning of modesty. By contrast, only 49% of child participants reported understanding its meaning, which did not differ significantly from chance (50%), *p* = .908. Children’s age was positively associated with their reported modesty understanding, *B* = 1.20, *OR* = 3.32, 95% CI [2.00, 5.49], *p* < .001. To provide a clearer picture of the development of modesty understanding, we report additional descriptive details for the child sample below. Children who correctly understood modesty were significantly older (*n* = 36; 16 girls and 20 boys; *M*_age_ = 9.62 years, *SD*_age_ = 1.25 years, age range = 6.80 to 11.16 years) than those who did not (*n* = 56; 19 girls and 19 boys; *M*_age_ = 7.59 years, *SD*_age_ = 1.13 years, age range = 5.96 to 10.59 years).

The age-specific patterns (using integer age) showed a clear developmental progression (see Table B3.5), with reported modesty understanding increasing markedly from age 9 years onward. By contrast, adults exhibited near-ceiling explicit reports of modesty understanding (98%), substantially higher than the child rate of 49%. These patterns indicate that explicit awareness of modesty emerges gradually across middle to late childhood and is largely consolidated by adulthood.

**Table B3.5**

*Modesty Understanding by Age in Children (Study 2)*

| Integer Age  (Years) | Reported  Modesty  Understanding | Counts | % of Total | Cumulative % |
| --- | --- | --- | --- | --- |
| 5 | No | 1 | 1% | 1% |
|  | Yes | 0 | 0% | 1% |
| 6 | No | 11 | 15% | 16% |
|  | Yes | 2 | 3% | 19% |
| 7 | No | 13 | 18% | 36% |
|  | Yes | 4 | 5% | 42% |
| 8 | No | 9 | 12% | 54% |
|  | Yes | 3 | 4% | 58% |
| 9 | No | 2 | 3% | 61% |
|  | Yes | 10 | 14% | 74% |
| 10 | No | 2 | 3% | 77% |
|  | Yes | 14 | 19% | 96% |
| 11 | No | 0 | 0% | 96% |
|  | Yes | 3 | 4% | 100% |

We relied on the forced-choice protagonist selections as our primary measures of understanding, as they more directly reflected participants’ context-specific comprehension of the constructs.

Across the full sample (*N* = 125), 73% of participants correctly identified the dishonest protagonist (honesty understanding), while 80% of those who made a selection (*N* = 123) correctly identified the modest protagonist (modesty understanding). Among children (*n* = 76), 59% answered the honesty item correctly (41% incorrectly) and—after excluding the two “I don’t know” responses—77% answered the modesty item correctly (23% incorrectly). Among adults (*n* = 49), 94% answered the honesty item correctly (6% incorrectly) and 84% answered the modesty item correctly (16% incorrectly).

Among children only, we tested whether continuous age in years predicted comprehension accuracy using separate logistic regressions. Age did not significantly predict accuracy on honesty understanding (*B* = 0.13, *p* = .374, *OR* = 1.14, 95% CI [0.85, 1.54]). However, age significantly predicted accuracy on modesty understanding (*B* = 0.58, *p* = .007, *OR* = 1.79, 95% CI [1.17, 2.72]), indicating that older children were more likely to correctly identify the modest protagonist.
